# Accurate characterization of dynamic microbial gene expression and growth rate profiles

**DOI:** 10.1093/synbio/ysac020

**Published:** 2022-10-15

**Authors:** Gonzalo Vidal, Carolus Vitalis, Macarena Muñoz Silva, Carlos Castillo-Passi, Guillermo Yáñez Feliú, Fernán Federici, Timothy J Rudge

**Affiliations:** Institute for Biological and Medical Engineering, Schools of Engineering, Biology and Medicine, Pontificia Universidad Católica de Chile, Santiago, Chile; Interdisciplinary Computing and Complex BioSystems (ICOS) Research Group, School of Computing, Newcastle University, Newcastle Upon Tyne, UK; Institute for Biological and Medical Engineering, Schools of Engineering, Biology and Medicine, Pontificia Universidad Católica de Chile, Santiago, Chile; Institute for Biological and Medical Engineering, Schools of Engineering, Biology and Medicine, Pontificia Universidad Católica de Chile, Santiago, Chile; Institute for Biological and Medical Engineering, Schools of Engineering, Biology and Medicine, Pontificia Universidad Católica de Chile, Santiago, Chile; School of Biomedical Engineering and Imaging Sciences, King’s College London, St Thomas’ Hospital, London, UK; Millennium Institute for Intelligent Healthcare Engineering (iHEALTH), Santiago, Chile; Interdisciplinary Computing and Complex BioSystems (ICOS) Research Group, School of Computing, Newcastle University, Newcastle Upon Tyne, UK; Department of Chemical and Bioprocess Engineering, School of Engineering, Pontificia Universidad Católica de Chile, Santiago, Chile; Institute for Biological and Medical Engineering, Schools of Engineering, Biology and Medicine, Pontificia Universidad Católica de Chile, Santiago, Chile; ANID – Millennium Science Initiative Program, Millennium Institute for Integrative Biology (iBio) & FONDAP Center for Genome Regulation, Santiago, Chile; Interdisciplinary Computing and Complex BioSystems (ICOS) Research Group, School of Computing, Newcastle University, Newcastle Upon Tyne, UK

**Keywords:** Inverse problem, characterization, dynamical systems, web application, gene expression

## Abstract

Genetic circuits are subject to variability due to cellular and compositional contexts. Cells face changing internal states and environments, the cellular context, to which they sense and respond by changing their gene expression and growth rates. Furthermore, each gene in a genetic circuit operates in a compositional context of genes which may interact with each other and the host cell in complex ways. The context of genetic circuits can, therefore, change gene expression and growth rates, and measuring their dynamics is essential to understanding natural and synthetic regulatory networks that give rise to functional phenotypes. However, reconstruction of microbial gene expression and growth rate profiles from typical noisy measurements of cell populations is difficult due to the effects of noise at low cell densities among other factors. We present here a method for the estimation of dynamic microbial gene expression rates and growth rates from noisy measurement data. Compared to the current state-of-the-art, our method significantly reduced the mean squared error of reconstructions from simulated data of growth and gene expression rates, improving the estimation of timing and magnitude of relevant shapes of profiles. We applied our method to characterize a triple-reporter plasmid library combining multiple transcription units in different compositional and cellular contexts in *Escherichia coli*. Our analysis reveals cellular and compositional context effects on microbial growth and gene expression rate dynamics and suggests a method for the dynamic ratiometric characterization of constitutive promoters relative to an *in vivo* reference.

**Graphical Abstract** 
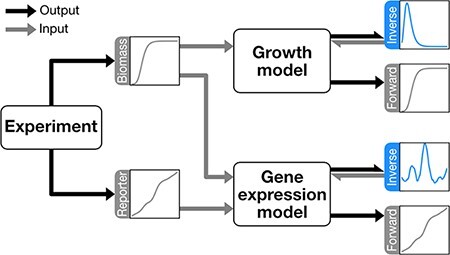

## 1. Introduction

Gene expression and growth are subject to variation due to changes in environmental and internal conditions, which may be divided into cellular and compositional contexts.

The cellular context is related to the cell or chassis itself such as the strain and to operational conditions such as media and carbon source. Microbial populations themselves create intrinsically dynamic conditions which can be separated into distinct phases of the growth cycle. An initial lag phase of adaptation is followed by exponential growth and finally a minimal growth stationary phase (1). These transitions in growth phases are driven by cell internal changes caused by depletion of nutrients, accumulation of waste products or due to biochemical and physical signals  (1). These signals can be part of the environment that the cell senses and responds to and can be experimentally determined.

Cellular context changes the metabolic activity of the cell and leads to distinct phenotypes (2). The effects of changes in external factors on growth and gene expression levels have been extensively studied (3–5) and correlations between them established. For example, ribosome and RNA polymerase are positively correlated with peak growth rate in different media (6). However, changes in the dynamics of gene expression due to cellular context are less clear (7), since different genes are known to be expressed differentially in each growth phase. This is at least partly due to the regulation of distinct sigma factor RNA polymerase sub-units, such as *σ*_70_ which peaks in exponential growth phase (8), as well as potentially gene-specific transcription factors, ribosome numbers and other translation factors (9).

The compositional context is related to the composition or sequence of the inserted DNA and can be seen at the transcription unit (TU), plasmid or genomic level. Synthetic biologists separate DNA into standard parts with determined sequences and function  (10). A composition of parts that enables transcription is a TU, and the transcribed RNA can be then translated into proteins. A TU capable of gene expression can be assembled with the parts promoter (Pro), ribosome-binding site (RBS), coding sequence (CDS) and terminator (Ter) in that exact composition order. The behavior of these parts varies depending on the surrounding sequences. While the magnitude of gene expression from a TU is known to be influenced by its surrounding sequences, orientation or its position relative to other parts in a plasmid or in the genome, their effect on dynamics is not well understood (11–14).

Measuring and analyzing the dynamics of gene expression are thus fundamental to understanding cellular regulation by both natural and synthetic gene networks in the face of different cellular and compositional contexts. Typical experiments to measure gene expression rates in bacteria and other microorganisms utilize fluorescent reporters to track the expression levels of lineages of cells (7,15–17). The total biomass of these lineages is also tracked, typically using optical density measurements or colony size (18, 19). The genes measured are often fusions of promoters of interest with a downstream fluorescent reporter, and their expression rate profiles are taken to be indicative of the transcription rate of the promoter (20, 7). Promoters have been characterized relative to a standard promoter (20–22) that is measured under the same conditions as the promoter of interest. This approach has been used under steady-state conditions but has not been applied to dynamics. Thus, there is a need for methods to characterize the dynamics of gene expression and growth as phenotypic parameters.

Reconstructing dynamic microbial gene expression and growth rate profiles from data is difficult because, particularly at low biomass, measurements suffer from significant noise. Typical methods involve data smoothing and differentiation of the resulting signal—herein referred to as the indirect method  (23–26). We consider two data smoothing filters; an anti-causal zero-phase digital filter (27) and the Savitzky–Golay polynomial filter (28). The indirect methods are sensitive to noise leading to the development of a more robust method based on the linear inversion of differential Equation models (29)—herein referred to as the direct method. Inverse problems present an approach to infer the values of parameters or functions on which measurements depend (30). The basic requirements to solve them are appropriate measurements and a mathematical model of the process that generates them. Two difficulties in inverse problems are that it cannot determine correlated parameters and that most problems of interest are ill-posed. An ill-posed problem is one that does not satisfy one or more of the well-posed properties: a solution exists, it is unique and its behavior changes continuously with the initial conditions. However, regularization methods, basis transformations and constraints can often be used to transform ill-posed into similar well-posed problems.

We present a method for the reconstruction of gene expression and growth rate profiles using inverse problems that achieves several times lower error than the direct and indirect methods. Our approach more accurately reproduced features of dynamic gene expression and growth rate profiles, including the lag phase and peak rates. Using this method, we characterized the dynamics of a collection of synthetic TUs in different cellular and compositional contexts relative to an *in vivo* reference, revealing uncertainty due to the genetic composition and external environmental factors. Our results suggest an approach to ratiometric characterization of the dynamics of gene expression.

## 2. Results

### 2.1 Gene Expression and Growth Dynamics

The rate at which a protein is synthesized by a genetic network or circuit varies over time, giving rise to dynamic gene expression rate profiles, which may include rich behaviors such as bistability (31) and oscillations (32). To see the importance of measuring these gene expression rate profiles, consider a genetic circuit composed of regulatory proteins. In the typical case of short half-life mRNAs, we may assume quasi-steady state and,


(1) $$ \frac{\mathrm{d}p_i}{\mathrm{d}t} = \phi_i(p_0, p_1, ..., p_{N-1}, t) - \gamma_i p_i - \mu(t)p_i, $$

for a genetic circuit with *N* proteins at concentrations *p*_*i*_, with degradation rates *γ*_*i*_, in cells growing at rate $\mu(t)$. The functions *ϕ*_*i*_ define the interactions in the circuit by mapping the protein concentrations to protein synthesis or gene expression rates. The dependence on time is due to variation in the cellular context such as growth phase transitions, which cause observable changes in the growth rate profile $\mu(t)$. It is, therefore, essential for the analysis of genetic circuit operation to estimate the gene expression rate profiles *ϕ*_*i*_ and growth rate profile *µ*, allowing the parameterization of models such as those in Equation 1.

The simplest genetic circuits consist of constitutive TUs which do not regulate each other, in which case $\phi_i(t)$ is only a function of time. To model the growth and reporter gene expression measurements from such a circuit in a population of cells, we use the following equations:


(2) $$ \frac{\mathrm{d}B}{\mathrm{d}t} = \mu(t) B, $$

(3) $$ \frac{\mathrm{d}y_i}{\mathrm{d}t} = B(t)\phi_i(t) - \gamma_i y_i, $$

where *B* is a measure of sample biomass, $\mu(t)$ is the instantaneous relative growth rate, *y*_*i*_ is the intensity of reporter, $\phi_i(t)$ is the instantaneous expression rate and *γ*_*i*_ is the reporter degradation rate for TU *i*. Here we have assumed that reporter intensity $y_i = Bp_i$, with *p*_*i*_ the protein concentration per biomass. While this is a reasonable assumption in constant conditions (33), this relationship may not always hold leading to inaccuracy in the reconstructed profiles, which is a general problem with gene expression reporters. More complex measurement models might be constructed, given sufficient information about the transformation of gene expression level into reporter intensity. We leave this for future work. From these equations we wish to accurately and robustly estimate the growth rate $\mu(t)$ and the expression rates $\phi_i(t)$, given an estimate of the reporter degradation rates *γ*_*i*_. Typically, reporter proteins are stable and so in the following we will use $\gamma_i=0$ (34) (see Supporting Information, Figure S1 for the effect of underestimating *γ*_*i*_).

Note that the expression rate $\phi(t)$ is different from the rate of change of fluorescence $\mathrm{d}y/\mathrm{d}t$ and from the rate of change of fluorescence concentration $\mathrm{d}(y/B)/\mathrm{d}t$, which both depend on protein degradation and dilution due to growth (and may be negative). What is being estimated in this work is the underlying fluorescent protein synthesis rate $\phi(t)$ (strictly positive), which is independent of dilution and degradation processes and which we assume is proportional to the underlying cellular gene expression rate (see Discussion).

### 2.2 Dynamics Estimation as an Inverse Problem

**Figure 1.**
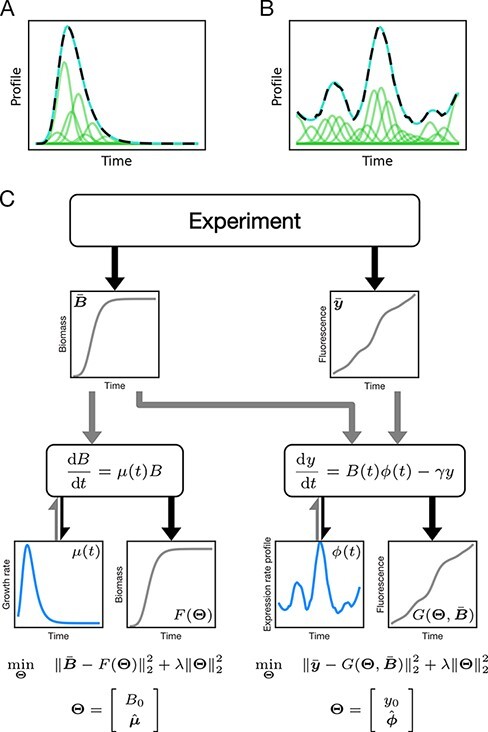
Algorithm overview. A, B Growth and gene expression rates approximation using Gaussian basis. The light green curves are individual Gaussian curves that compose the basis. The turquoise line represents the sum of the Gaussian basis. The black dashed line represents the growth rate profile (A) or gene expression rate profile (B) to be fitted. (C) Inverse problem algorithm diagram. Once an experiment is performed, biomass data ($\bar{\boldsymbol{B}}$) and fluorescence data ($\bar{\boldsymbol{y}}$) are collected. These data are input to the models, where the biomass model requires only biomass as input and the fluorescence model requires both biomass and fluorescence. Finally, growth rate ($\mu(t)$) and expression rate ($\phi(t)$) profiles are generated from the biomass and fluorescence models, respectively. The gray arrows indicate inputs and black arrows indicate outputs.

Reconstructing the functions $\mu(t)$ and $\phi(t)$ represents an inverse problem, which is underdetermined and ill posed (30). In order to reduce the dimensionality of the inverse problem, we exploit prior knowledge of the functions $\mu(t)$ and $\phi(t)$ to construct a simple basis as follows. Expression rates and growth rates may be reasonably assumed to be strictly positive and smooth on typical timescales of transcription and translation. We propose the following approximation, given a function *f*(*t*) that meets our assumptions,


(4) $$ f(t) \approx \sum_{k=0}^{n-1} \hat{f}_k G_k(t){,} $$

with


(5) $$ G_k(t) = \mathrm{exp} \left( \frac{-(t-k\Delta)^2}{2\Delta}\right), $$

which represents a sum of *n* Gaussian curves *G*_*k*_ with weight $\hat{f}_k$, variance Δ and regularly spaced over time *t* at intervals Δ. Here Δ determines the timescale of variation or smoothness of the representation of the function *f*(*t*). Since here we consider bulk culture experiments, gene expression bursting and noise in growth cannot be observed, and the relevant timescales are the gene expression reporter half-life and the culture doubling time, typically <1 h. Choosing Δ greater than the sampling interval of the data makes the system over-determined and regularized in the sense that it is constrained to be smooth. In the following we choose $\Delta=1$ h, the typical timescale of protein synthesis, which is larger than the usual sampling interval of 10–15 min (see Methods). In Figure 1A and B we can see examples of reconstructed growth rate and gene expression rate profiles in turquoise and the function to be fitted in dashed black. The effect of this approximate Gaussian basis can be seen from the dependence on Δ of the maximum slope of the basis functions $G_k(t)$, which scales as $1/\sqrt{\Delta}$. This means that the sharpest change in expression rate and growth rate profiles that can be reconstructed by our method is determined by Δ (see Supporting Information, Figure S2 for effect of Δ on reconstruction error).

The model given in Equations 2 and 3 combined with the approximation of Equations 4 and 5 represents the forward models of the inverse problems for the reconstruction of $\mu(t) \approx \sum_k \hat{\mu}_k G_k(t)$ and $\phi(t) \approx \sum_k \hat{\phi}_k G_k(t)$. In practice, the measurements used to estimate *B* and *y* are discrete, will contain background signal and are subject to noise. The background signals *B*^ʹ^ and *y*^ʹ^ are typically estimated by measuring appropriate control samples containing no cells (*B*^ʹ^) and cells with no reporter expression (*y*^ʹ^) (see Methods). After subtracting these background measurements, we are left with the noisy estimates $\bar{\boldsymbol{B}}$ and $\bar{\boldsymbol{y}}$. We then wish to parameterize the forward models given by Equations 2 and 3 such that $\Vert\boldsymbol{y}-\bar{\boldsymbol{y}}\Vert_2^2$ and $\Vert\boldsymbol{B}-\bar{\boldsymbol{B}}\Vert_2^2$ are minimized.

Note that while this approach uses a smooth basis to represent the reconstructed profiles, it does not filter or smooth the input data as in the indirect method. Here we approximate the growth and gene expression rate profiles using a superposition of Gaussian functions to represent a continuous function as a discrete vector of parameters. The measurements and the estimated parameters are inputs of the forward model. The forward model then generates simulated measurements using different estimated parameters, and we compute the ones that minimize the difference between the measurements and the model.

### 2.3 Accurate Estimation of Growth Rate Dynamics

The inverse problem for the reconstruction of the growth rate $\mu(t)$ can be stated as,


(6) $$ \min_{\boldsymbol{\Theta}}\quad \Vert \bar{\boldsymbol{B}} - F(\boldsymbol{\Theta}) \Vert_2^2 + \lambda \Vert\boldsymbol{\Theta}\Vert_2^2 $$

with,


(7) $$ \boldsymbol{\Theta} = \left[\begin{array}{c} B_{0}\\ \hat{\boldsymbol{\mu}} \end{array}\right]. $$

We minimize the difference between noisy estimates of Biomass $\bar{\boldsymbol{B}}$ and the result of the forward model $F(\boldsymbol{\Theta})$. Since this problem is ill-posed we regularize using a Tikhonov penalty term *λ*. **Θ** is the vector of parameters to optimize, containing the initial biomass *B*_0_ and the Gaussian basis weights $\hat{\mu}_k$ to represent $\mu(t) \approx \sum_{k=0}^{n-1} \hat{\mu}_k G_k(t)$. The hyperparameter *λ* was chosen to minimize error within a reasonable range of values (see Supporting Information, Figure S2).

**Figure 2.**
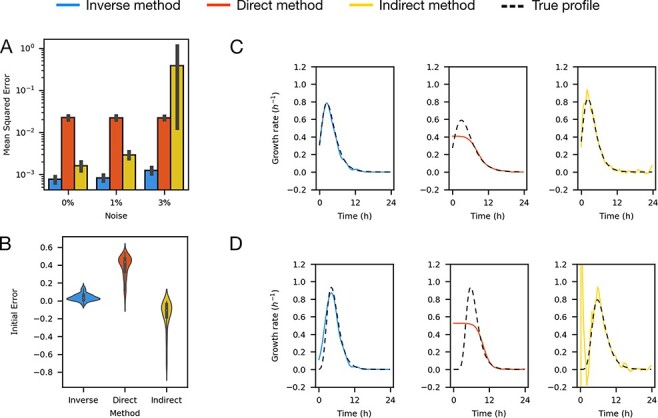
Method comparison on growth rate simulations. (A) The errors corresponding to the inverse, direct and indirect methods are presented, and the error of the inverse method was almost 30-fold lower than the direct method and 2-fold lower than the indirect method; error bars represent $95\%$ confidence interval. (B) The error of the initial growth rate over all noise levels. Growth rate, due to its nature, has a peak, which is not easily identified with the direct method. Since the initial growth rate should be low, we can see that the direct method overestimates and the indirect method underestimates the initial growth rate, showing that the inverse method is more accurate at low biomass. The error calculated for growth rate values at time 0 compared to the true profile. (C) Examples of accurate growth rate reconstructions from simulated data. Gompertz profiles characterized using the inverse, direct and indirect methods (left, center and right, respectively) compared to the true profile (black dashed line). **D** Examples of inaccurate growth rate reconstructions from simulated data. Gompertz profiles characterized using the inverse, direct and indirect methods (left, center and right, respectively) compared to the true profile (black dashed line) (*n* = 100).

This problem is a nonlinear least squares optimization, which we solve using the trust region reflective algorithm (35). We generated simulated data from Equations 2 and 3 using 100 randomly parameterized Gompertz growth models (1) and three different levels of measurement noise and characterized the growth rate using the inverse, direct and indirect methods (Supporting information Figures S3–S5). Hyperparameters for each method were optimized to minimize error within a reasonable range of values (see Supporting Information, Figure S2). We compare our results to the direct linear inversion method and show that our approach reduces mean squared error by more than 29-fold ($P\lt{10}^{-35}$, Welch’s *t*-test) (Figure 2A). Then we compared to the indirect method and show that the inverse method reduced the mean squared error by >2-fold ($P\lt{10}^{-5}$, Welch’s *t*-test) (Figure 2A). Finally, we compared to a different indirect method that uses an anti-causal zero-phase digital filter and show that the inverse method reduced the mean squared error by more than a 1000-fold (Supporting information Figure S17). The inverse method maintained the best performance at high noise levels. Furthermore, our method is more robust to noise in early biomass measurements, where the direct method overestimates the initial values and the indirect produces noisy reconstructions that usually goes below zero at the beginning (Figure 2B). The inverse method correctly reconstructed the lag phase, which is missing from the linear inversion solution. Our method also reconstructed better the growth rate peak, effectively distinguishing between lag, exponential and stationary growth phases (Figure 2C and D).

### 2.4 Accurate Estimation of Gene Expression Rate Dynamics

**Figure 3.**
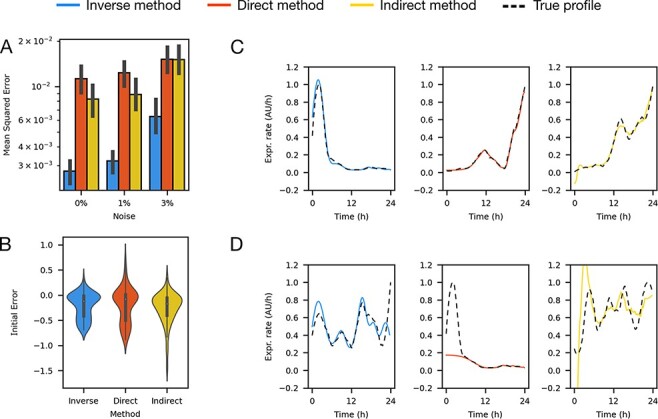
Method comparison on gene expression rate simulations. (A) The errors corresponding to the inverse, direct and indirect methods are presented; the inverse method has been shown to be 4-fold better than the direct method and close to 3-fold better than the indirect method, error bars represent $95\%$ confidence interval. (B) The error at initial time. Since the initial gene expression rate simulations were not restricted to be low at the beginning, the three methods show similar errors. The error calculated for expression rate values at time 0 compared to the true profile. (C) Representative examples of accurate gene expression reconstructions from simulated data. Random profiles characterized using the inverse, direct and indirect methods (left, center and right, respectively) compared to the true profile (black dashed line). (D) Representative examples of inaccurate gene expression rate reconstructions from simulated data. Random profiles characterized using the inverse, direct and indirect methods (left, center and right, respectively) compared to the true profile (black dashed line) (*n* = 100).

In a similar way to growth rate, we wish to find the optimal parameters,


(8) $$ \boldsymbol{\Theta}=\left[\begin{array}{c} y_{0}\\ \hat{\boldsymbol{\phi}} \end{array}\right], $$

where **Θ** is the vector of parameters to optimize, containing the initial reporter intensity *y*_0_ and the Gaussian basis with weights $\hat{\boldsymbol{\phi}}_k$ and the forward model $G(\boldsymbol{\Theta}, \bar{\boldsymbol{B}})$. The problem is again a nonlinear least squares optimization,


(9) $$ \min_{\boldsymbol{\Theta}}\quad \Vert \bar{\boldsymbol{y}} - G(\boldsymbol{\Theta},\bar{\boldsymbol{B}}) \Vert_2^2 + \lambda \Vert\boldsymbol{\Theta}\Vert_2^2 $$

which we solve using the same numerical procedure as for growth rate. To test this approach, we generated 100 random gene expression rate profiles from smoothed lognormal random walks, with random Gompertz models for the biomass, and three measurement noise levels (Supporting information Figures S6–S8) (see Methods). Again, we compared the inverse method to the direct linear inversion method and show that the mean squared error is more than 4-fold lower ($P\lt{10}^{-10}$, Welch’s *t*-test) and close to 3-fold lower than the indirect method ($P\lt{10}^{-7}$ Welch’s *t*-test) (Figure 3A). Finally, we compared to a different indirect method that uses an anti-causal zero-phase digital filter and show that the inverse method reduced the mean squared error by more than a 100-fold (Supporting information Figure S17). The gene expression simulations are not constrained to start with low values or to have an initial peak which make the three methods have similar initial errors (Figure 3B). We find that the direct method does not correctly reconstruct early peaks in gene expression rate profiles and that the indirect method produces extremely noisy solutions (Figure 3C and D). The inverse method is the best performer capturing the shape of the growth rate profile, although this method suffers with sharp changes due to its dependence with Δ (Figure 3C and D). The inverse method requires knowledge of the timescale of the process that you want to measure to set the hyperparameter Δ (see Supporting information).

### 2.5 Characterizing Growth and Gene Expression Rate Dynamics in *Escherichia coli*

**Figure 4.**
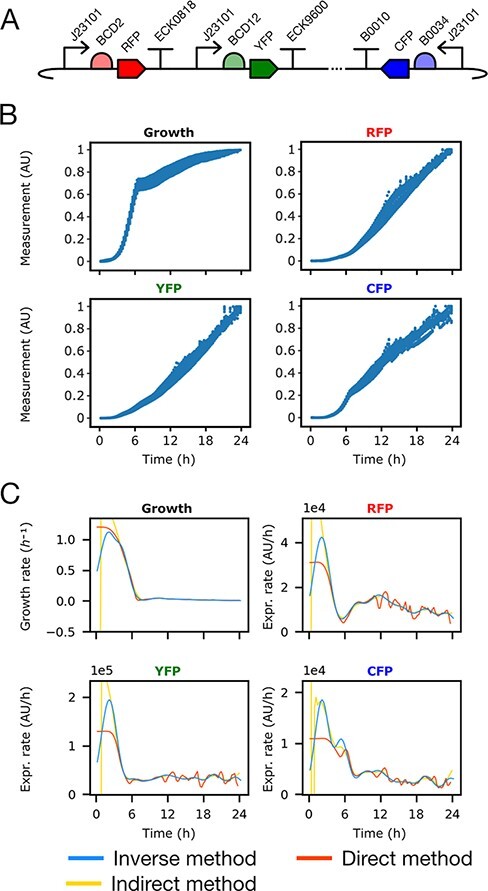
Gene expression dynamics shape reconstructed from experimental data is different for transcription units with the same promoter. (A) Plasmid pAAA synthetic biology open language (SBOL) visual diagram. (B) Raw experimental data of optic density (OD) and fluorescence. (C) Growth and gene expression rates reconstructed with the inverse, direct and indirect methods (blue, red and yellow lines, respectively) on strain MG1655z1 in media M9-glucose (*n* = 30).

Using the inverse, direct and indirect methods we reconstructed growth and gene expression dynamics from experimental data of *Escherichia coli* carrying a synthetic triple TU plasmid pAAA (Figure 4A and B). This plasmid contains three TUs with the same synthetic *σ*_70_ constitutive promoter J23101 (36) in different plasmid compositional contexts determined by its position in the plasmid and in different TU compositional contexts determined by different sets of promoter downstream elements RBS-CDS-Terminator (Figure 4A). Each CDS encoded a different fluorescent protein as reporter. Assays were performed using a 96-well microplate reader to measure fluorescence in each reporter channel as a proxy for protein concentration and optical density as a proxy for biomass (see Methods). To assess the effects of cellular context on growth and gene expression dynamics we measured two strains of *E. coli* carrying the pAAA plasmid, growing on two different carbon sources (26) (Figure 4B–E) (see Supporting information, Figure S9 for an example of the raw fluorescence and OD 600 data).

The inverse method captured the shape of the growth and gene expression rate profiles in a smooth way, consistent with population averages on the timescale of protein synthesis, while the direct and indirect methods produced noisy solutions. Our method reconstructed a peak in growth rate consistent with the transition between lag, exponential and stationary phases, as in the Gompertz growth model (37). Furthermore, it captured a peak in the expression rate coincident with the growth rate peak, which is consistent with promoter dependence on *σ*_70_ and the abundance of this factor, as well as ribosomes, during peak growth (Figure 4B–E) (6, 9).

The gene expression rate profiles were different for TUs in the same plasmid under the control of the same promoter due to both different cellular and compositional contexts (Figure 4B–E). While in all cases peak gene expression rate coincided with peak growth rate, in some contexts multiple peaks were observed. The cellular context (strain and carbon source) as well as the compositional context (promoter downstream elements and position and orientation in the plasmid) clearly change gene expression and growth dynamics, leading to different peak timing and overall shape. All growth rates characterized using the inverse method exhibited clear lag, exponential and stationary phases, which are not apparent with the direct method (Figure 4B–E). The timing of growth phase transitions was different in each cellular context (Figure 4B–E).

### 2.6 Characterization of Gene Expression Rate Dynamics Relative to *in vivo* Reference

Gene expression magnitude has been characterized relative to a standard *in vivo* reference containing promoter J23101, in order to normalize for cellular context (22). Plasmid pAAA provides such a reference, with three TUs containing the same J23101 promoter in different compositional contexts. We hypothesized that this reference plasmid could be used to characterize the dynamics of gene expression in a standardized fashion. Each TU in the pAAA plasmid presents a standard reference for a particular compositional context—the promoter downstream elements, position and orientation in the plasmid. We then wish to characterize TUs with arbitrary promoters relative to these reference TUs, allowing us to describe their dynamics in a concise way. In order to compare gene expression rate dynamics from different experiments we synchronized profiles and normalized each one by subtracting its mean and dividing by its standard deviation. The timing of growth phase transitions is variable due to differences in initial conditions and experimental variability, which leads to differences in the timing of gene expression rate profiles. In order to correct for these differences, we used the reconstructed growth rate peak time *t*_0_ to synchronize the expression rate profiles, shifting time to $\tau = t - t_0$, such that *τ* = 0 is the time of peak growth rate.

We tested this approach on a collection of 14 combinatorial three-reporter plasmids, combining 10 different TUs which were each driven by one of seven promoters (38). Each plasmid contained three TUs producing red fluorescent protein (RFP), yellow fluorescent protein (YFP) and cyan fluorescent protein (CFP), containing the same promoter downstream elements as the corresponding reference TU. The promoter downstream elements RBS, CDS and terminator for each reporter were maintained constant and we refer to them using the reporter name (Supporting information Tables S1 and S2). The CFP TU was maintained the same in all plasmids, to serve as a control (22, 20). This collection of plasmids presented a variety of promoters in different TU compositional contexts, that is, with different promoter downstream elements. Each TU was assembled into multiple plasmid compositional contexts, that is, in the presence of different upstream or downstream TUs.

**Figure 5.**
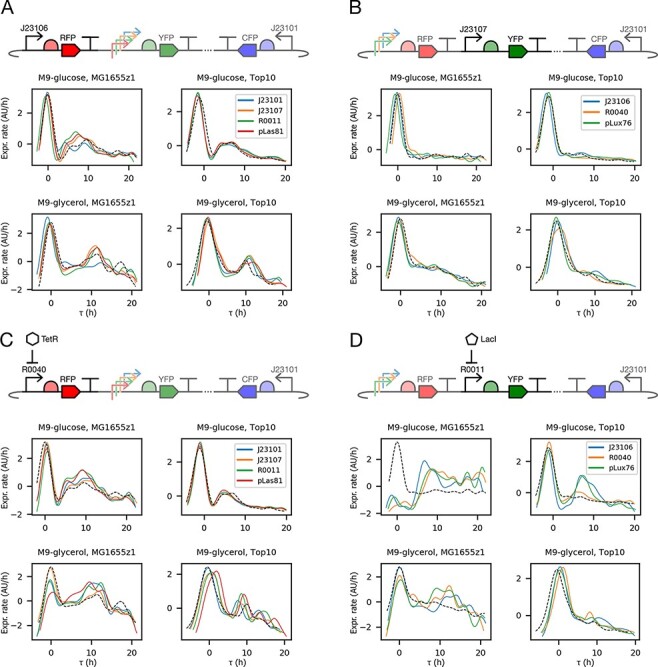
The gene expression profile shape reconstructed from experimental data is similar for promoters under constitutive expression. We used TUs from pAAA as reference (black dashed line) for TUs within the same conditions but with different promoters (solid lines). (A) RFP reference TU compared to RFP TU with J23106 in different downstream TU compositional contexts. (B) YFP reference TU compared to YFP TU with J23107 in different upstream TU compositional contexts. (C) RFP reference TU compared to RFP TU with R0040 basal expression in different downstream TU compositional contexts. (D) Reference YFP TU compared to YFP TU with R0011 with different upstream TUs compositional context. All profiles are shown normalized by mean and standard deviation (*n* = 30).

Three of the TUs in the collection contained a promoter from a family of constitutive promoters created by mutating a consensus sequence (36), which includes the reference TU promoter, J23101. The RFP reference TU matched the gene expression rate profile shape of the RFP TU with the constitutive promoter J23106 over different downstream TU contexts (Figure 5A). The YFP reference TU matched the gene expression profile shape of the YFP TU with the constitutive promoters J23107 (Figure 5B) and J23101 (Supporting information Figure S10B) over different upstream TU contexts. The CFP TU was consistent across all compositional contexts (Supporting information Figure S10A). These results suggest that the dynamics are not affected by the tested plasmid compositional contexts.

Two of the TUs contained promoters that are repressible by a transcription regulating protein that binds to the promoter. In the case of the TetR-responsive TU (containing promoter R0040), the RFP reference TU matched the gene expression rate profile over different downstream TU contexts and over different cellular contexts (Figure 5C). This is in spite of the fact that the genome of the strain MG1655z1 contains a constitutive TetR gene, while Top10 does not produce the protein. This result suggests that the dynamics of the TetR-repressible TU are not affected by the action of the repressor.

The other repressible TU contained promoter R0011, regulated by LacI. LacI is produced by both strains and partly regulated by cAMP in Top10 (39). The YFP reference TU matched the LacI-repressible YFP TU gene expression rate profiles in M9-glycerol but did not match them in M9-glucose (Figure 5D). In MG1655z1 growing on glucose gene expression rates became negatively correlated with growth rate, and in Top10 on glucose they exhibited a second peak. These results suggest that transcription regulation can significantly affect the dynamics of gene expression compared to constitutive expression profiles.

The remaining two TUs contained promoters activated by different one-component signaling systems, measured in the absence of signal and regulator to study their basal expression. The RFP TU responsive to C6 homoserine lactone, containing promoter pLux76, was consistent with the constitutive reference TU in all contexts (Supporting information Figure S10C). However, for the YFP TU responsive to C12 homoserine lactone, containing promoter pLas81, the gene expression rate profile inverted its correlation with growth rate when downstream of the TU containing promoter R0040 and growing on glucose in strain MG1655z1. All the magnitudes of these measurements can be found in Supporting information Figure S13. These results show the uncertainty that can be introduced in gene expression rate dynamics due to changing compositional and cellular context (Supporting information Figure S10D).

## 3. Discussion

The accurate characterization of dynamic gene expression and growth rate profiles is essential for the characterization of genetic circuits and the inference of gene regulatory interactions in natural networks (40–42). We have demonstrated an inverse problem approach to reconstructing dynamic gene expression rate and growth rate profiles from noisy kinetic measurement data. We compared our method to the current state-of-the-art algorithms, direct linear inversion (29) and indirect smoothing and differentiation (25). Our approach reduced the mean squared error of reconstructions from simulated data of growth rate by almost 30-fold and gene expression rate by more than 4-fold with respect to the direct method.

The comparison showed that the direct method often fails to capture peaks at the beginning of the profiles. Indirect methods, even after filtering the noise, fail to reconstruct early stages of growth. This is likely because the biomass can have very low and even negative values after background correction and dividing by these values can result in the amplification of noise. This highlights that particular attention should be paid to reducing noise from experimental procedures and measurement techniques since all methods attain lower reconstruction errors with less noise. Surprisingly, our indirect method often performed better than the direct method, but our inverse problems approach improved on the indirect method by almost 3-fold for gene expression rates, and more than 2-fold for growth rate. Furthermore, we were able to reconstruct features of both growth rate and gene expression rate profiles, such as exponential phase and peak growth, that were not apparent from the direct linear inversion method nor the indirect method. The growth rate peak is an important feature captured better with our method, allowing the synchronization of gene expression rate profiles.

While in terms of computation time our method is relatively slow, it is the most intuitive to adjust by estimating the timescale of dynamics to obtain Δ. The indirect methods require knowledge of the signal processing filters used, and the direct method requires tuning the ‘insignificant value’ which is rather obscure.

Using our method we showed that the dynamic form of gene expression rates, not only their magnitude, is determined both by cellular and compositional contexts (Figure 5, Supporting information Figures S10 and S13–S15). We examined two types of compositional context. Firstly, the composition of parts within a TU, not only the sequence of the promoter, determined the dynamics of gene expression rates. Secondly, gene expression rate profiles were largely independent of the context in which the TU was placed, that is the upstream and downstream TUs. In most cases, gene expression rates peaked in exponential growth phase, with some promoters exhibiting a second peak in stationary phase. This may be an effect of the abundance of different sigma factors in each growth phase and the sensitivity of the promoter to them. It is known that the binding sites for *σ*_70_, most abundant in exponential growth phase, and *σ*_*S*_, most abundant in stationary growth phase, are very similar (43). Therefore, a promoter with peaks in both exponential and stationary phases may be activated by both *σ*_70_ and *σ*_*S*_ to different extents and the peaks caused by variations in the sigma factor abundance in each growth phase. In some cases gene expression rate profiles inverted their correlation with growth rate, highlighting the uncertainty introduced by changing circuit composition. This uncertainty may be due to various mechanisms that modulate gene expression at sequence level such as DNA supercoiling (12).

Furthermore, our results also showed that cellular context, that is media and strain, changed dynamic gene expression and growth rate profiles. Our results suggest that while the dynamic characterization for TUs under constitutive or leaky expression relative to an *in vivo* reference could be useful, uncertainty due to compositional and cellular context must be taken into account. This highlights the need for strategies to mitigate compositional context effects, such as gene expression load (44), five prime untranslated region (45) as well as techniques to predict interactions of genetic elements at the sequence level (11,21,46).

Typically, measurements of promoter–reporter fusions are used as a proxy for transcription rates under various levels of transcription factors, external signals and other determinants of gene expression (20,21,47,48). However, applying our method to multiple reporter TUs with the same promoter, we showed that gene expression rate profiles should not be taken as indicative of intrinsic characteristics of promoters, since they are affected by both the promoter and downstream genetic elements, gene position and orientation and external factors such as carbon source and host strain (see Supporting information Figure S16).

Synthetic biology aims to design novel genetic networks or circuits from compositions of transcription units. It relies heavily on the characterization of the functions of these TUs. Fundamentally, gene expression rates must be reconstructed from noisy measurement data in a range of conditions, including concentrations of inducer chemicals. The function of the circuit may then be mathematically modeled as in Equation 1. Methods such as ours will enable such approaches for dynamical systems (32, 31) where the dynamic profile of gene expression rates is essential to the operation of the circuit.

In order to model circuit operation in this way, calibration of the fluorescence and biomass signals with respect to standard references (15, 18) or the use of relative (ratiometric) quantification (21, 20) are necessary and can be easily incorporated into our workflow. Our results show that profiles for constitutive gene expression were consistent across a range of promoters in a range of contexts suggesting that an *in vivo* reference gene may be used to infer the expression profiles of other genes in a circuit without directly measuring them. The inverse problems approach provides a framework that could be extended easily to fit more complex models of gene expression and also for regulatory parameters (e.g. Hill functions) as well as dynamic profiles, providing an accurate and flexible characterization method for synthetic biology.

## 4. Methods

### 4.1 Kinetic Gene Expression and Growth Assays

Kinetic gene expression and growth assays were made culturing triple reporter plasmid pAAA containing bacteria (Figure 4A) using two different strains as well as two different carbon sources, as described in Flapjack (26).

Monoclonal colonies of *E. coli* strain TOP10 or MG1655z1 transformed with plasmid DNA were picked and cultured for 14–15 h overnight in M9 media with 50 $\mu$g/ml kanamycin, 0.2% w/v casaminoacids and 0.4% w/v glucose or 0.4% w/v glycerol. Overnight cultures were diluted 1000 times in 2-ml tubes. All the tubes were filled with 1996µ l of fresh M9 media, 2µ l of kanamycin and 2µ l of the bacteria liquid culture obtaining a final volume of 2 ml. In each well of a 96-well plate were added 200 µ l, 4 wells with M9 media with the proper carbon source and kanamycin, 4 wells with non-transformed bacteria of the same strain and 10 wells of bacteria transformed with the appropriate plasmid to analyze from the previously prepared 2-ml tubes. Optical density and fluorescence in three channels (RFP, YFP and CFP) were measured approximately every 15 min for 24 h in a Synergy HTX plate reader with Gen5 software. Each assay was repeated on three different days, with 10 replicates on each day, and each 96 well plate contained experiments with the same carbon source and strain following the methods in Flapjack (26).

### 4.2 Computational Methods

Computational methods were performed using Flapjack (26), Python (49), Numpy (50), Scipy (51), Pandas (52), Matplotlib (53), Plotly (54), Jupyter (55), Google Colaboratory  (56) and Matlab. The direct method was computed using the WellFARE package (29). The indirect method with anti-causal zero-phase digital filter was computed using the Matlab function ‘butter’ (27).

### 4.3 Web-based Software Implementation

Flapjack (26) (http://flapjack.rudge-lab.org) was extended to compute gene expression rate and growth rate profiles using the methods described above, using the WellFARE package (29) for the direct method. The indirect method is computed by Flapjack by filtering the measured signals (biomass and reporter levels) using a Savitzky–Golay filter (28) and then differentiating the resulting smooth polynomial interpolation.

Flapjack is a systems and synthetic biology data storage and analysis tool, built as a web app that provides a user-friendly web interface and a representational state transfer (REST) and web socket application programming interface (API). The system allows upload of kinetic gene expression data from a variety of sources and links it to metadata about experimental conditions. These data may then be queried and filtered and used to reconstruct gene expression and growth rates. Flapjack automatically subtracts background signal from both reporter and biomass measurements, taking the average of control samples (untransformed cells or media with no cells) at each time point.

### 4.4 Random Profile Generation

In order to generate a range of gene expression rate profiles, with minimal assumptions about their form, we generate lognormal random walks as,


(10) $$ \phi_t=\prod_{i=0}^{t}\xi_i $$

with $\mathrm{log} (\xi_i) \sim N(0, \sigma^2)$ and $\sigma^2=0.25$. The profiles *ϕ*_*t*_ were then smoothed using a second-order Savitzky–Golay filter (28) with window size 21 and normalized to $[0,1]$. To generate random growth rate profiles, we used the Gompertz equation,


(11) $$ \mathrm{log}\left(\frac{B}{B_0}\right) = A\ \mathrm{exp}\left(-\mathrm{exp}\left(\frac{\mu^*\mathrm{e}}{A} (\lambda-t) + 1 \right)\right) $$

with $\mu^*$ the maximal growth rate uniformly distributed on $[0.5, 1]$ per hour, *λ* the lag phase length uniformly distributed in $[0,4]$ hours, $A = \mathrm{log}(B^*/B_0)$, where the maximal biomass $B^*=1$ and minimum biomass $B_0=0.01$. The growth rate profile implied by this equation is given by,


(12) $$ \mu(t) = \mu^* \mathrm{exp} \left[ \frac{\mu^* \mathrm{e}\cdot (\lambda - t)}{A} - \ \mathrm{exp}\left(\frac{\mu^*\mathrm{e}\cdot (\lambda - t)}{A} + 1 \right) + 2 \right]. $$

### 4.5 Simulation of Kinetic Gene Expression and Biomass Data

Equations 2 and 3 were solved using the forward Euler integration scheme with time step $\Delta t = 2.4 \times 10^{-2}$ h for a period of 24 h. Noise and background were added according to the following equations with $B^{\prime}=0.1$ and $y^{\prime}=0.1$,


(13) $$ B_t = (B(t) + B^{\prime}) (1 + \epsilon_t) $$

(14) $$ y_t = (y(t) + y^{\prime}) (1 + \zeta_t),$$

where *ϵ*_*t*_ and *ζ*_*t*_ are uncorrelated white noise with variance *σ*^2^, due to the measurement process. Simulated measurements were generated using LOICA (57), then uploaded to Flapjack (26) and analyzed using the API via Python (see Supporting information Figures S11 and S12 for an example of reporter and biomass raw data, respectively).

### 4.6 Choice of hyperparameters

Each of the methods tested in the main text is dependent on one or more hyperparameters. In the case of the direct method this is the so-called insignificant value *ϵ*_*L*_ (29), for the indirect method it is the Savitzky–Golay filter window size and for the inverse method they are Δ and *λ*. For reconstructions of simulated data these parameters were optimized by scanning a range of reasonable values and choosing the parameter that minimized the mean squared error (see Supporting information). For experimental data, the value of *λ* was chosen using the L-curve method (58), *ϵ*_*L*_ was taken from the original paper (29), the value of Δ was fixed at 1 h and the Savitzky–Golay window size fixed at 11. For the anti-causal zero-phase digital filter, a second-order Butterworth filter (27) with cut-off frequency 4/33 was used.

## Supplementary Material

ysac020_Supp

## Data Availability

The data are available at https://github.com/RudgeLab/Inverse_Characterization/tree/main/Data.
