# Supplementary material for: Accurate characterization of dynamic microbial gene expression and growth rate profiles
Source: Synth Biol (Oxf). 2022 Oct 15;7(1):ysac020. doi: 10.1093/synbio/ysac020 (PMC9569155; doi:10.1093/synbio/ysac020)
Supplement: ysac020_Supp [file ysac020_supp.zip › suppl_data/pub_OUP_supp.pdf]

OUP Synthetic Biology, 2022, pp. 1–19

doi: DOI HERE
Advance Access Publication Date: Day Month Year
Supplementary Information

SUPPLEMENTARY INFORMATION

Accurate Characterization of Dynamic Microbial Gene Expression and Growth Rate Profiles

Gonzalo Vidal,[1,4] Carlos Vidal-Céspedes,[1] Macarena Muñoz Silva,[1] Carlos Castillo-Passi,[1,5,6] Guillermo Yáñez Feliú,[2,4] Fernán Federici[1,3] and Timothy J. Rudge[4,*]

[1]Institute for Biological and Medical Engineering, Schools of Engineering, Biology and Medicine, Pontificia Universidad Católica de Chile, Avda.Vicuña Mackenna, 7820244, Santiago, Chile, [2]Department of Chemical and Bioprocess Engineering, School of Engineering, Pontificia Universidad Católica de Chile, Avda.Vicuña Mackenna, 7820244, Santiago, Chile, [3]ANID – Millennium Science Initiative Program, Millennium Institute for Integrative Biology (iBio) & FONDAP Center for Genome Regulation, Avda. Libertador Bernardo O'Higgins , 8331150, Santiago, Chile, [4]Interdisciplinary Computing and Complex BioSystems (ICOS) Research Group, School of Computing, Newcastle University, NE4 5TG, Newcastle Upon Tyne, U.K., [5]School of Biomedical Engineering and Imaging Sciences, King's College London, St Thomas' Hospital, SE1 7EH, London, U.K. and [6]Millennium Institute for Intelligent Healthcare Engineering (iHEALTH), Santiago, Chile
[*]Corresponding author. tim.rudge@ncl.ac.uk
FOR PUBLISHER ONLY Received on Date Month Year; revised on Date Month Year; accepted on Date Month Year

Abstract

Gene expression model

A simple two-step transcription-translation model of gene expression dynamics can be formulated as [1]:

$$\frac{\mathrm{d}m}{\mathrm{d}t} = k_{TX}(t) - \delta m \tag{1}$$

$$\frac{\mathrm{d}p}{\mathrm{d}t} = mk_{TL}(t) - \gamma p - \mu(t)p \tag{2}$$

with m the mRNA concentration, p the reporter protein concentration, $k_{TX}(t)$ and $k_{TL}(t)$ the transcription and translation rates, δ and γ the corresponding degradation rates of mRNA and protein, and $\mu(t)$ is the instantaneous relative growth rate. In the typical case of short half-life mRNAs, we may assume quasi-steady state and,

$$\frac{\mathrm{d}p}{\mathrm{d}t} = \phi(t) - \gamma p - \mu(t)p \tag{3}$$

with,

$$\phi(t) = \frac{k_{TX}(t)k_{TL}(t)}{\delta} \tag{4}$$

Thus, the expression rate $\phi(t)$ of the reporter depends on both transcription and translation rates. Both of these rates are time varying and regulated in response to changing environmental conditions [2].

Optimization of hyperparameters for reconstruction from simulated data

The hyperparameters were chosen based on the difference between the true profile and the method using those hyperparameters (see Methods). The selected hyperparameters minimize the MSE between the reconstructed and true profiles (See Figure 2). For the inverse method, an exploration of values for Δ -ranging from 0.5 to 2, with 10 intermediate points- was performed, finding that the optimum value for growth rate corresponded to 1.3, while for expression rate it was 1.5. We also explore the values of λ -ranging from 10^{-4} to 10^{0}, with 10 intermediate points log-spaced- finding that the optimum value for growth rate correspond to 0.016681, while for expression rate it was 0.005995. For the direct method, an exploration of values for the insignificant value (ϵ_L) -ranging from 10^{-8} to 10^{-2}, with 10 intermediate points log-spaced- was performed, finding that the optimum value for growth rate corresponded to 2.154×10^{-7}, while for expression rate it was 10^{-6}. For the indirect method, an exploration of values of the window size or smooth was performed with the following values: [9, 11, 13, 15, 17, 19]. The optimum value for growth rate found corresponded to 11, while for expression rate it was 17.

In the case of Δ, to estimate the maximal possible slope of reconstructions we differentiate equation (9) (main text) and find analytically that the slope is $\frac{1}{\sqrt{e}\sqrt{\Delta}}$.

© The Author 2022. Published by Oxford University Press. All rights reserved. For permissions, please e-mail: journals.permissions@oup.com

Table 1. Table indicating the different symbols used for each promoter for easy reference.

Symbol	Promoter
A	J23101
B	J23106
C	J23107
D	R0011
E	R0040
F	pLas81
G	pLux76

Table 2. Table showing the components of the constructs used for each reporter. RBS, CDS, and Terminator are indicanted.

Reporter	RBS	CDS	Terminator
RFP	BCD2	mRFP	ECK0818
YFP	BCD12	EYFP	ECK9600
CFP	B0034	ECFP	B0015

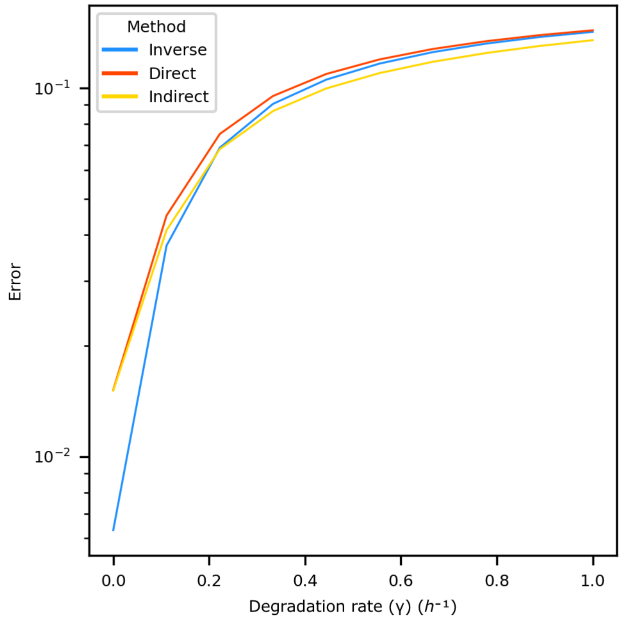

Fig. 1. Effect of underestimating the degradation rate (γ). Simulations were performed with a range of degradation rates, and reconstructions were made assuming degradation rate $\gamma = 0$.

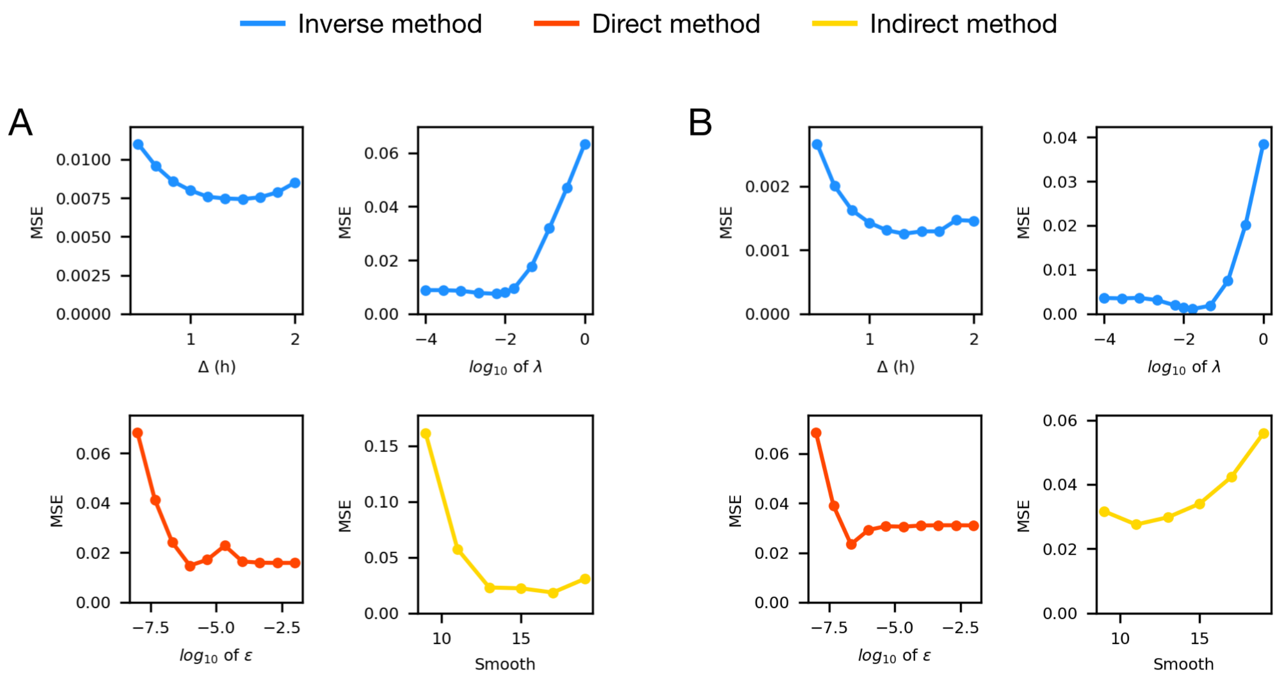

Fig. 2. An exploration of the hyperparameters corresponding to the different methods was carried out in order to reduce the error of each one. **A** Plots of the values obtained from the exploration of a range of values for each hyperparameter corresponding to each method in gene expression profiles. **B** Plots of the values obtained from the exploration of a range of values for each hyperparameter corresponding to each method in growth expression profiles.

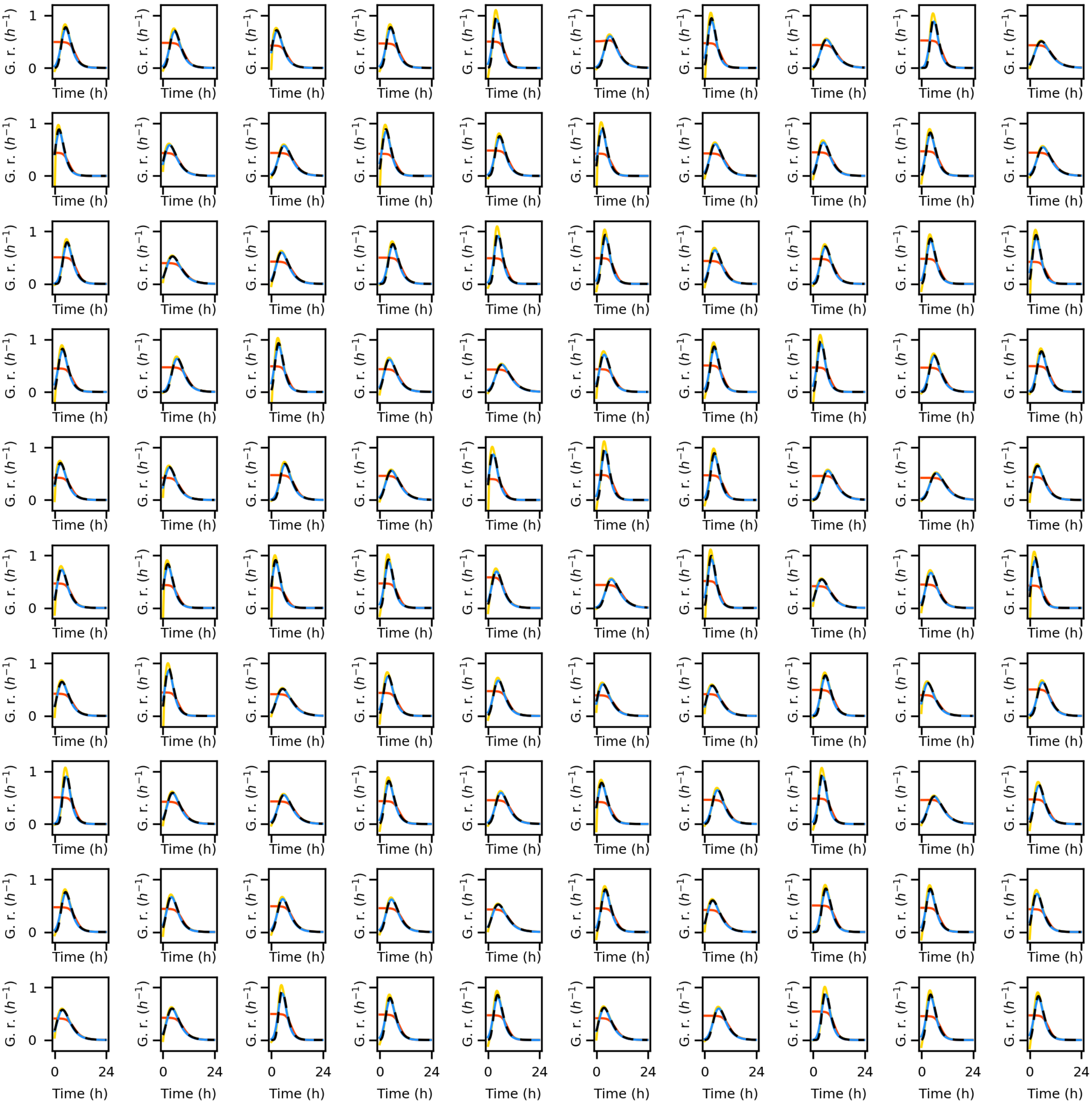

Fig. 3. All 100 growth rate reconstructions from simulated data for Gompertz profile with no noise. The direct method (red line) overestimates initial growth rate and does not reconstruct the peak while the indirect method has minor difficulties to correctly represent the first expression interval, whereas the inverse method (blue line) reproduces the true profile (black dashed line) in a smoother manner.

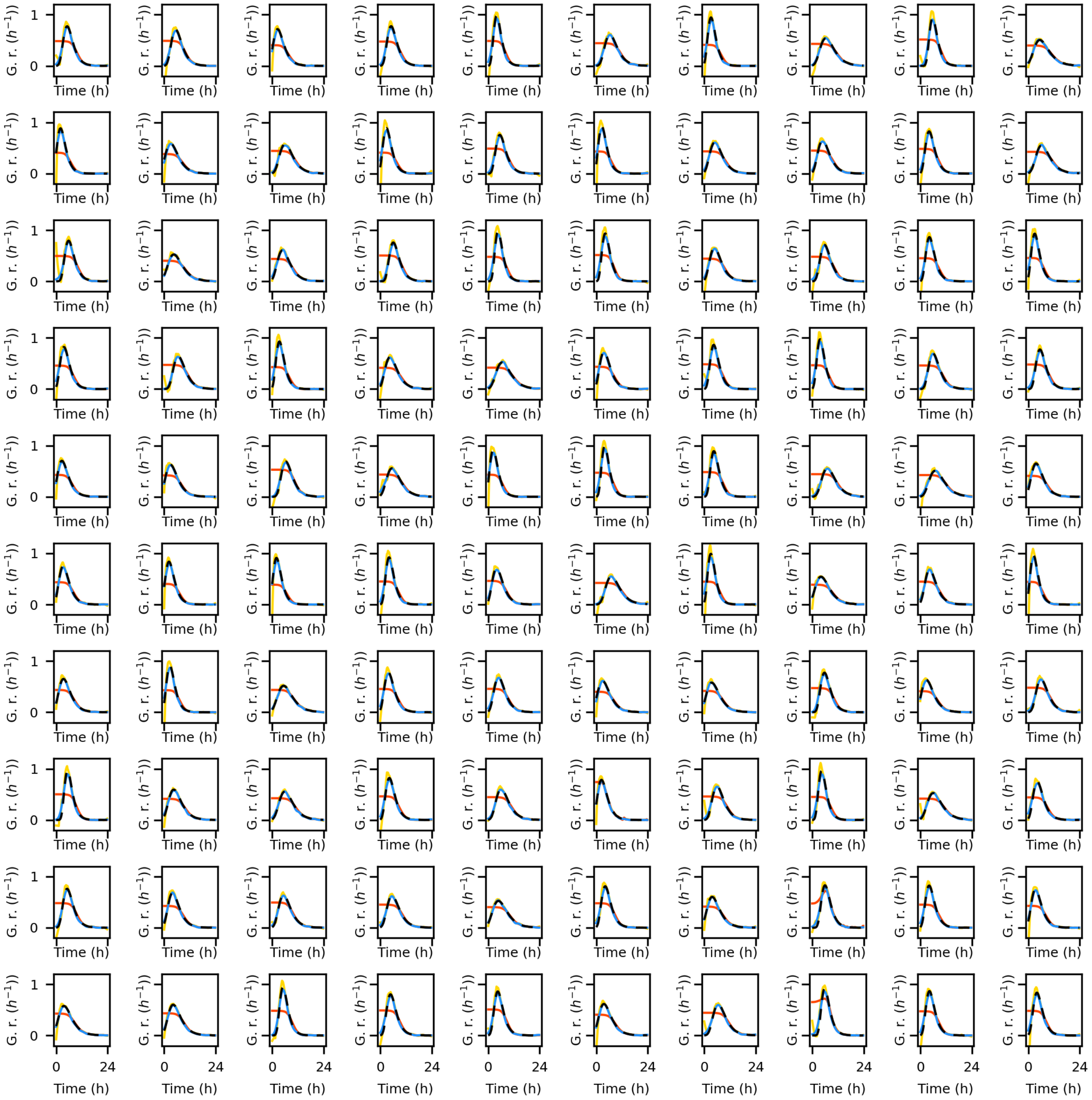

Fig. 4. All 100 growth rate reconstructions from simulated data for Gompertz profile with 1% noise. The direct method (red line) overestimates initial growth rate and does not reconstruct the peak while the indirect method has minor difficulties to correctly represent the first expression interval, whereas the inverse method (blue line) reproduces the true profile (black dashed line) in a smoother manner.

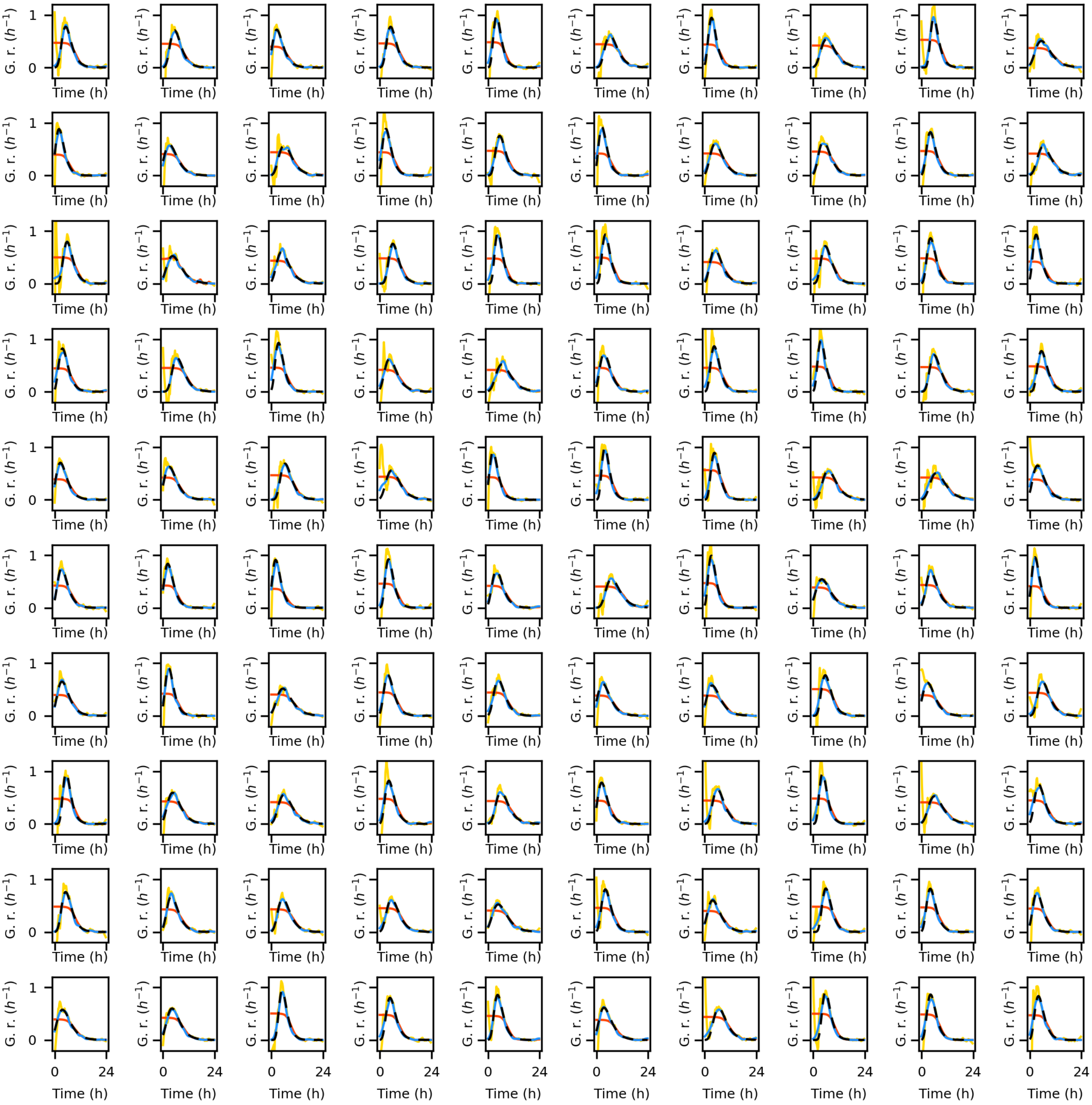

Fig. 5. All 100 growth rate reconstructions from simulated data for Gompertz profile with 3% noise. The direct method (red line) shows some noisy reconstructions while the indirect method has difficulties to correctly represent the first expression interval, whereas the inverse method (blue line) reproduces the true profile (black dashed line) in a smoother manner.

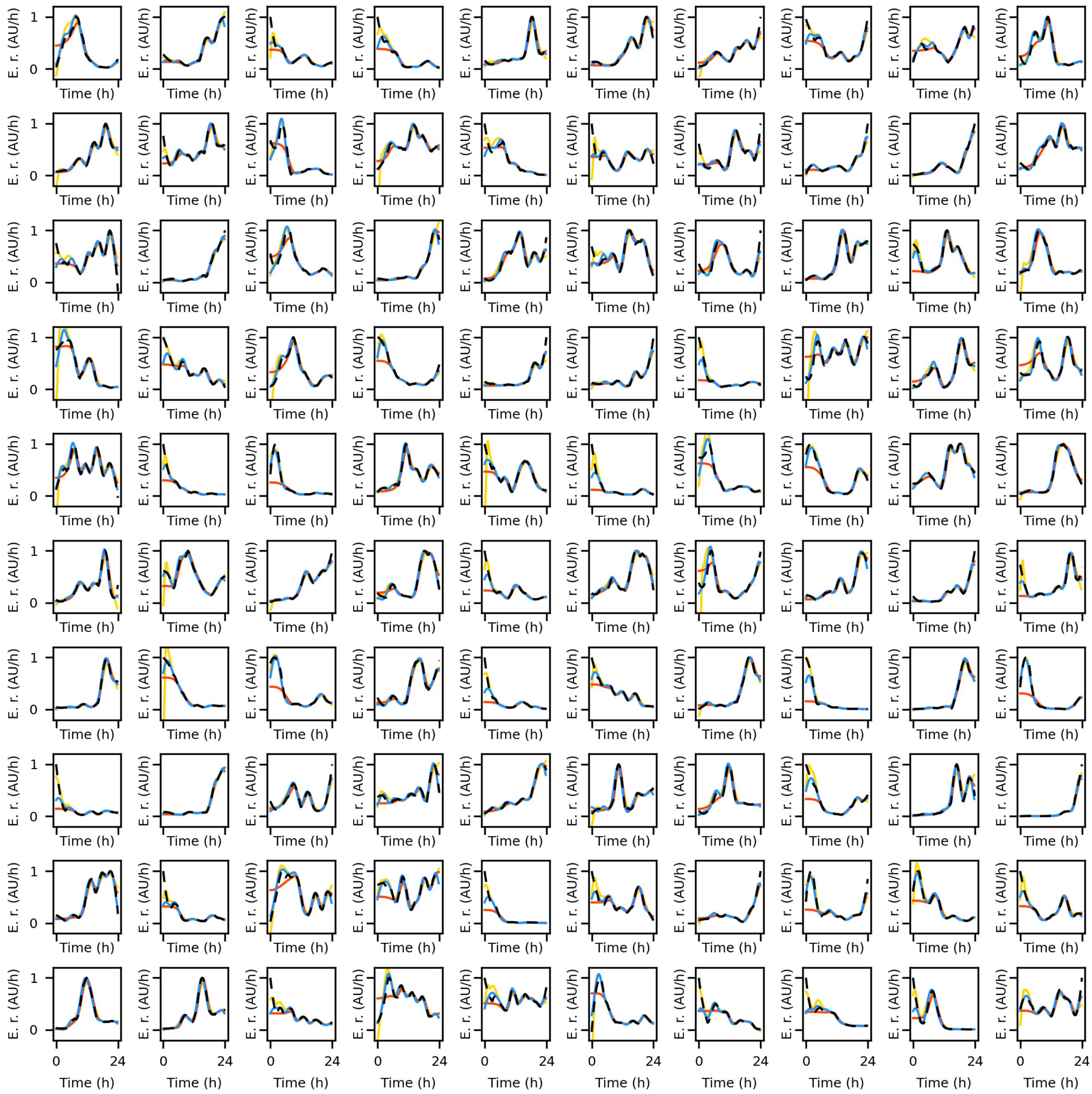

Fig. 6. All 100 expression rate reconstructions from simulated data for Brownian profile with no noise. The direct method (red line) shows some noisy reconstructions while the indirect method has difficulties to correctly represents the first expression interval, whereas the inverse method (blue line) reproduces the true profile (black dashed line) in a smoother manner.

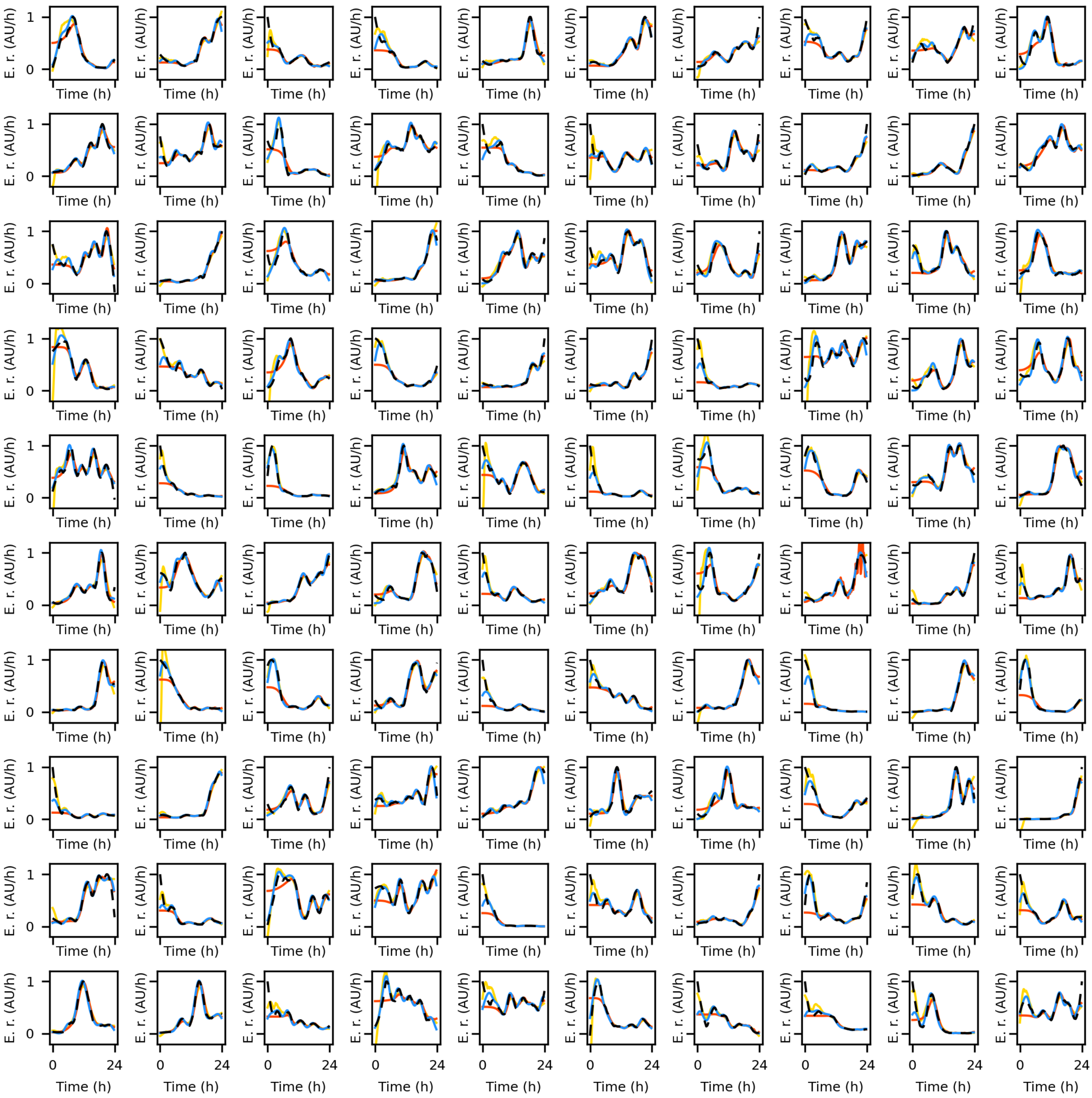

Fig. 7. All 100 expression rate reconstructions from simulated data for Brownian profile with 1% noise. The direct method (red line) shows some noisy reconstructions while the indirect method has difficulties to correctly represent the first expression interval, whereas the inverse method (blue line) reproduces the true profile (black dashed line) in a smoother manner.

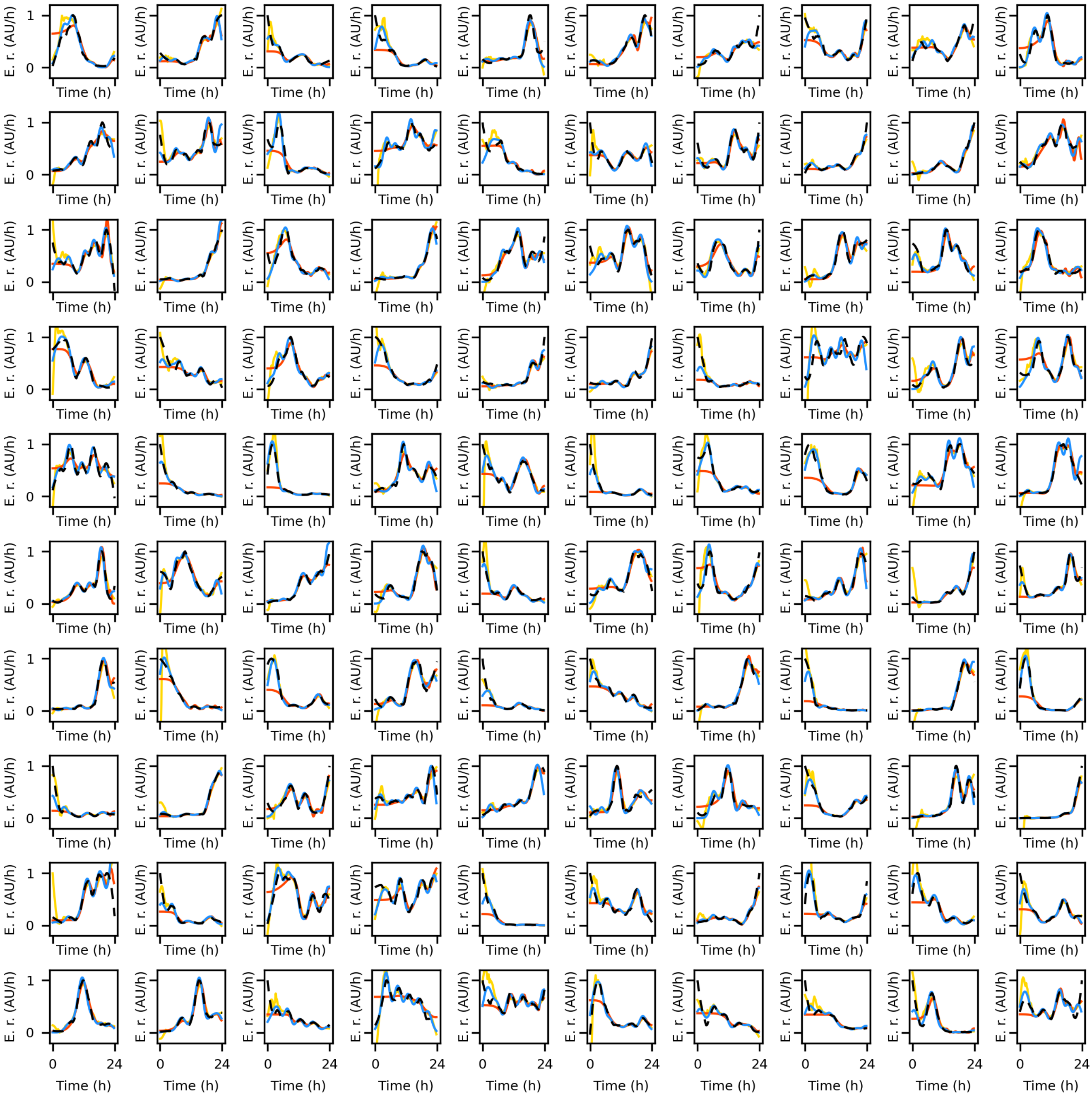

Fig. 8. All 100 expression rate reconstructions from simulated data for Brownian profile with 3% noise. The direct method (red line) shows some noisy reconstructions while the indirect method has difficulties to correctly represents the first expression interval, whereas the inverse method (blue line) reproduces the true profile (black dashed line) in a smoother manner.

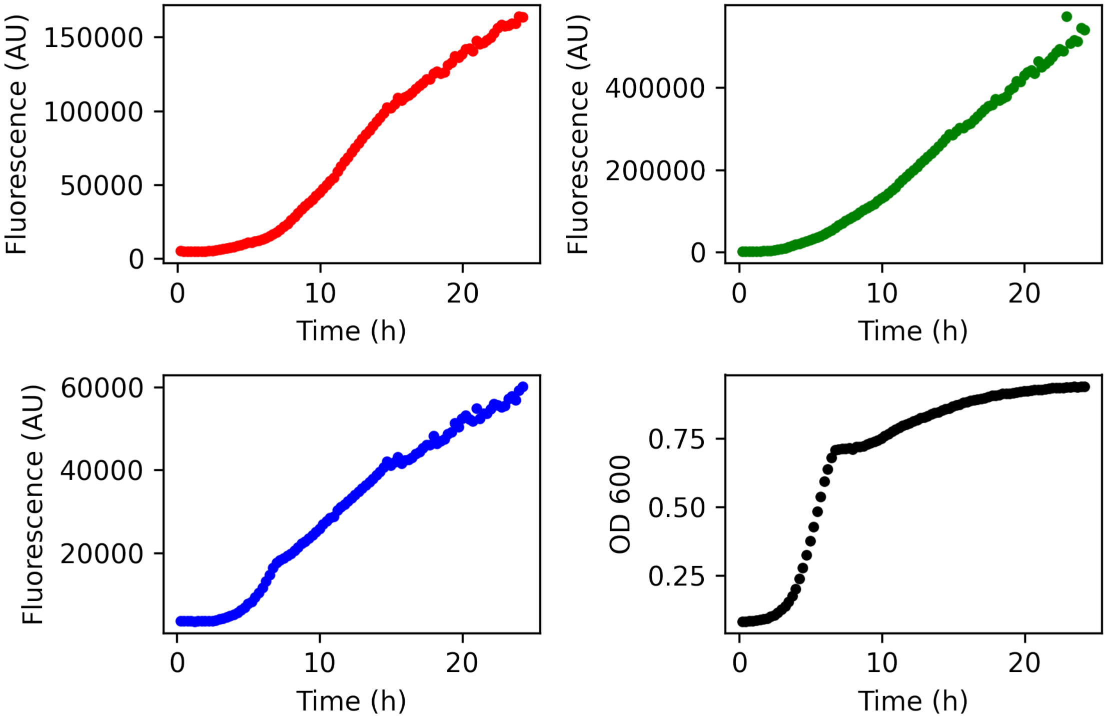

Fig. 9. Experimental pAAA raw data. Example of fluorescence data of the reporters RFP, YFP, and CFP and OD 600 data of biomass.(n=1)

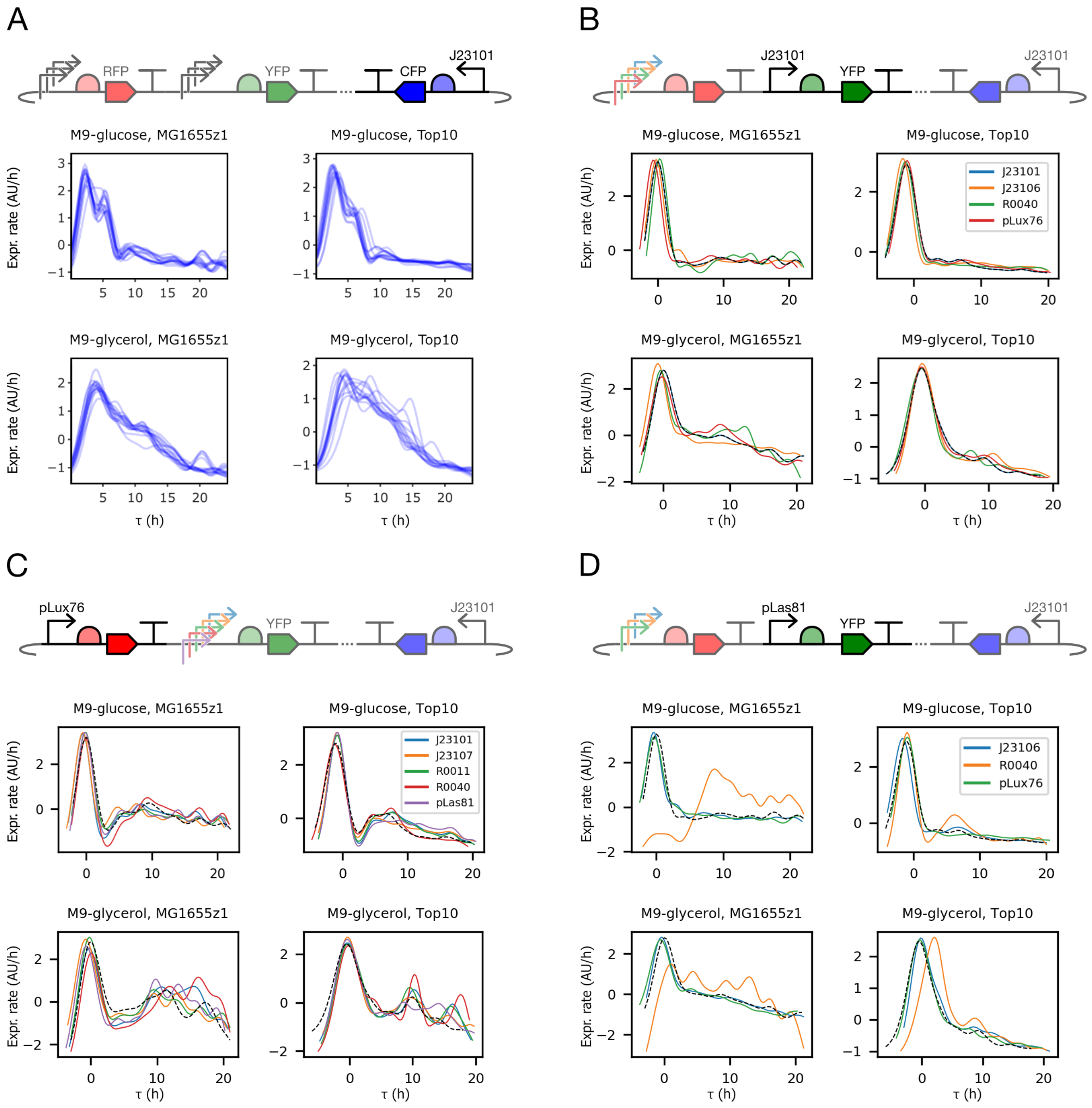

Fig. 10. The gene expression profile shape reconstructed from simulated data is similar for promoters under constitutive expression. **A** Characterization of all CFP TUs in different plasmid compositional contexts splited by cellular context. We used TUs from pAAA as reference (black dashed line) for TUs within the same conditions but with different promoters. **B** YFP reference TU compared to YFP TU with J23101 in different upstream TUs compositional contexts. **C** RFP reference TU compared to RFP TU with pLux76 basal expression in different downstream TUs compositional contexts. **D** YFP reference TU compared to YFP TU with pLas81 with different downstream TUs compositional context. As expected, the reference captures the profile of TUs with its J23101 promoter. The reference also captures the inducible promoters pLux76 and pLas81 profiles. The only profile not captured by the reference corresponds to pLas81 with R0040 in the upstream TU showing variance produced by compositional context.(n=30)

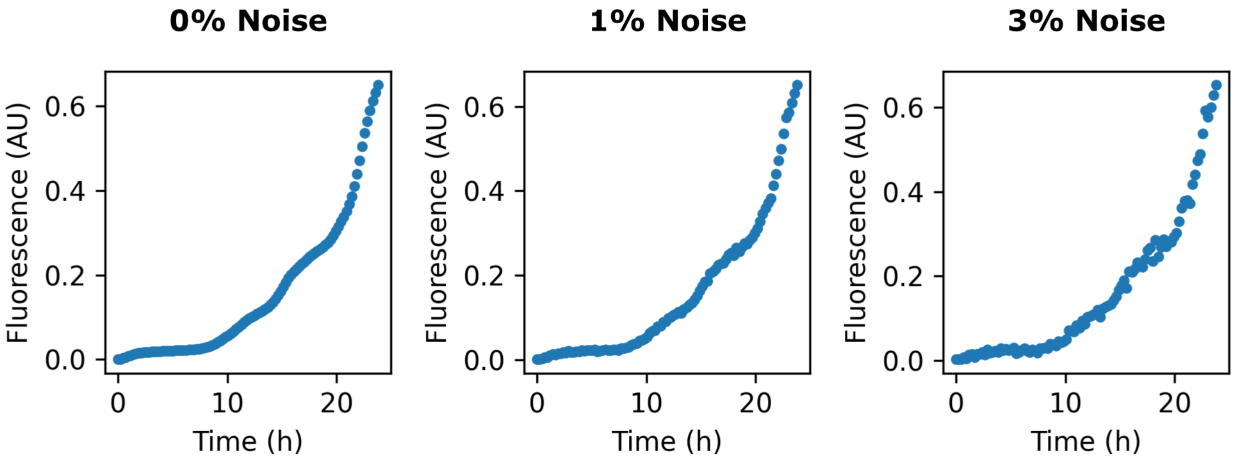

Fig. 11. Simulated reporter raw data. Synthetic reporter data for the reconstruction of random profiles generated with different noise levels, 0%, 1% and 3%. (n=1)

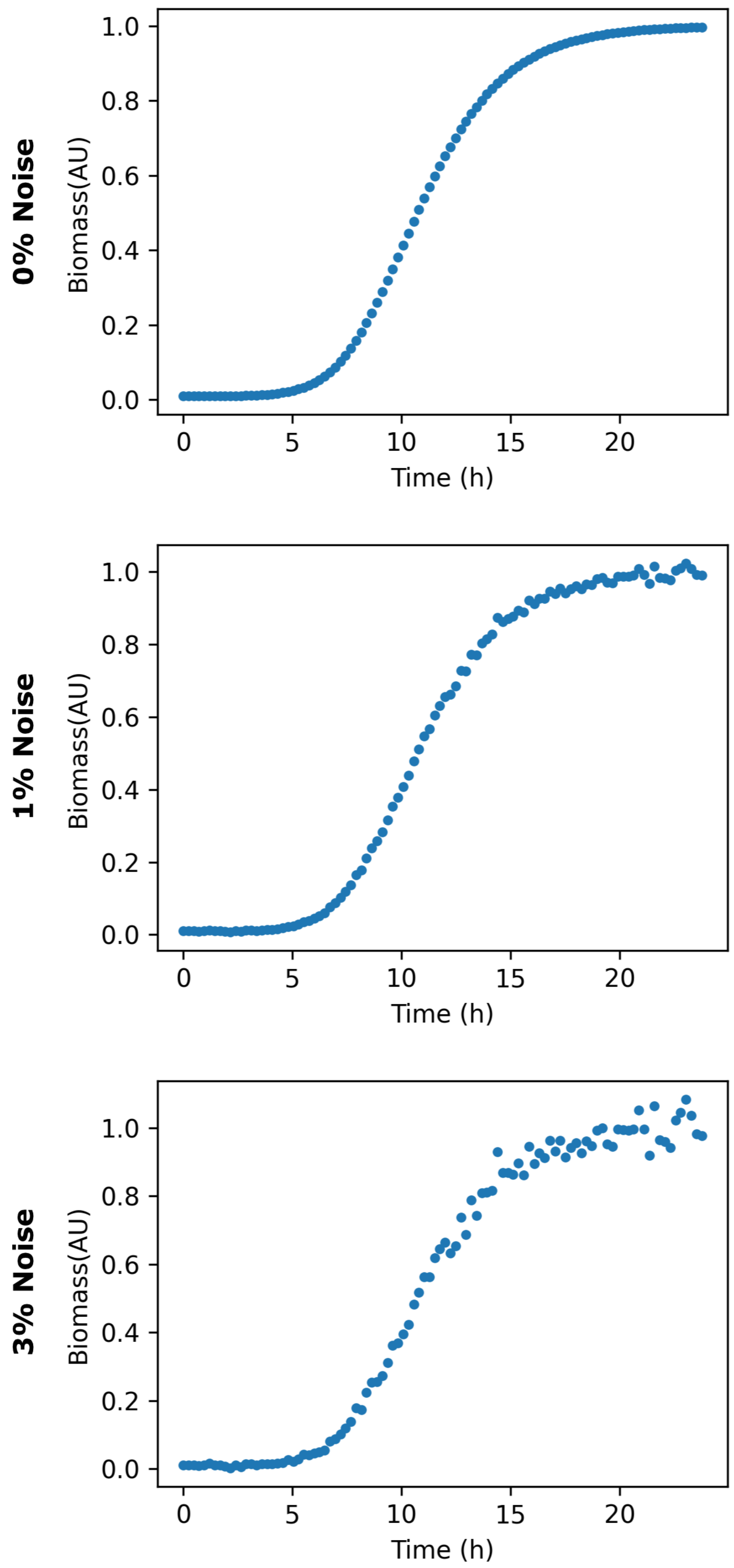

Fig. 12. Simulated biomass raw data. Synthetic biomass data generated using the Gompertz model with different noise levels, 0%, 1% and 3% from top to bottom. (n=1)

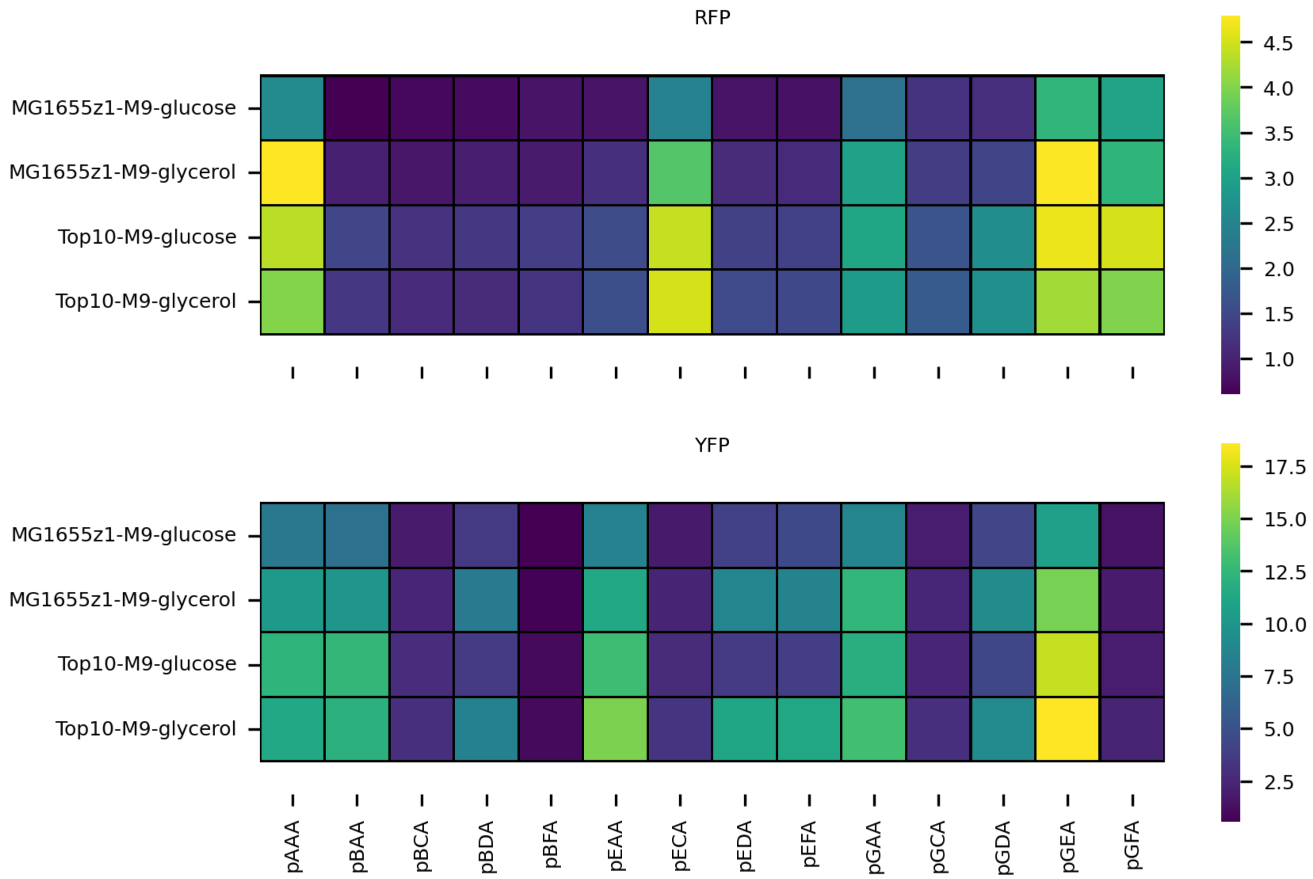

Fig. 13. Relative mean expression heatmap of experimental data. Mean expression of each signal for all 14 plasmids were obtained and RFP and YFP where divided by its corresponding CFP value to obtain relative fluorescent units.(n=30)

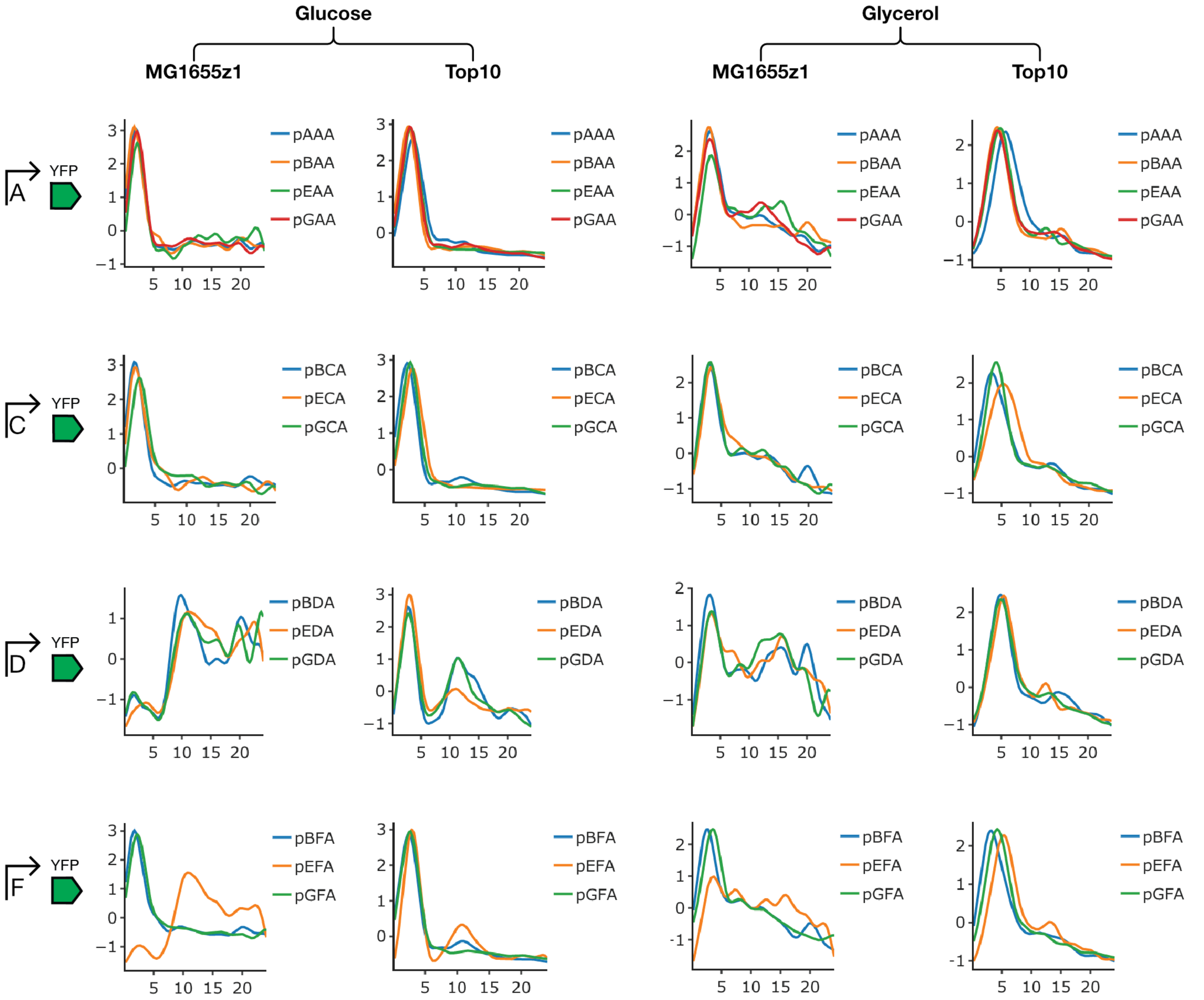

Fig. 14. Experimental data of YFP reporter expression in different compositional contexts and conditions. Graphs were grouped according to the promoter they possess to appreciate the effect of conditions on expression. The Glycerol MG1655z1 column presents the expressions with the greatest differences compared to the rest, illustrating the role of conditions in expression. The particular case of the F promoter in the pEFA construct stands out, which shows a different expression from the other constructs using the same promoter when found in the MG1655z1 medium, regardless of the strain.(n=30)

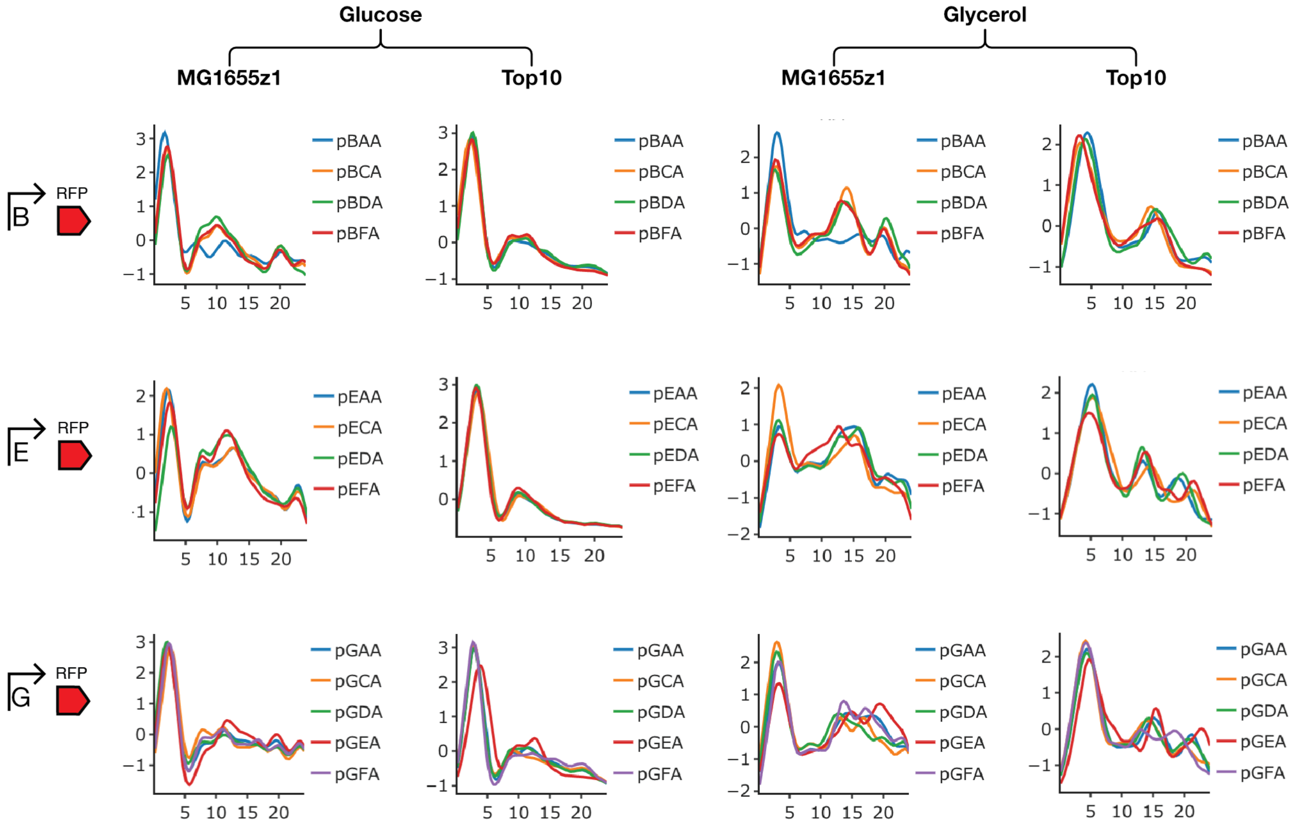

Fig. 15. Experimental data of RFP reporter expression in different compositional contexts and conditions. Graphs were grouped according to the promoter they possess to appreciate the effect of conditions on expression. The Glycerol MG1655z1 column presents the expressions with the greatest differences compared to the rest, illustrating the role of conditions in expression.(n=30)

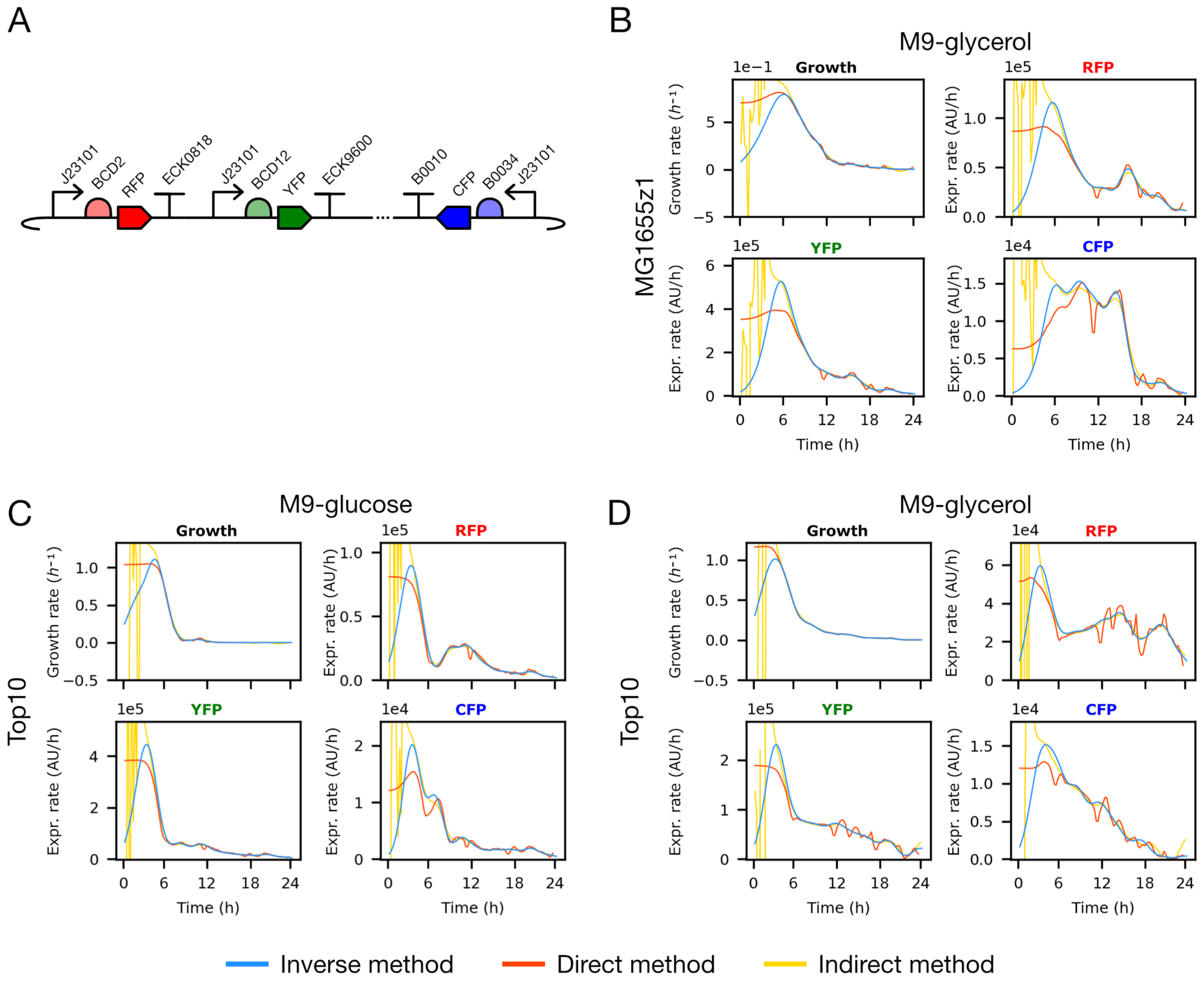

Fig. 16. Gene expression dynamics shape reconstructed from experimental data is different for transcription units with the same promoter. **A** Plasmid pAAA SBOL visual diagram. Growth and gene expression rates reconstructed with the inverse, direct and indirect methods (blue, red and yellow lines respectively) on different combinations of strain and carbon source: **B** M9-glycerol, MG1655z1; **C** M9-glucose, Top10; **D** M9-glycerol, Top10. (n=30)

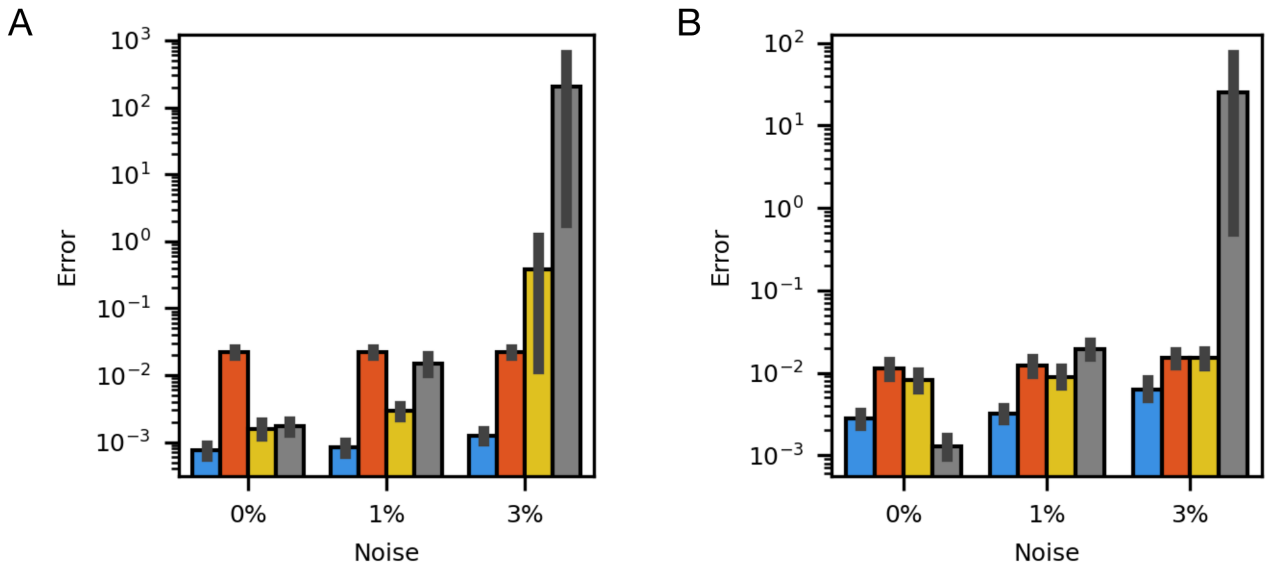

Fig. 17. Methods comparison with indirect with anti-causal zero-phase digital filter method. Simulations of growth (A) and expression rate (B) where reconstructed with different noise levels. The errors corresponding to the inverse (blue), direct (red), indirect (yellow) and indirect with anti-causal zero-phase digital filter (gray) methods are presented, the error of the inverse method was almost 30-fold lower than the direct method, two-fold lower than the indirect method and more than a thousand times lower than the indirect with anti-causal zero-phase digital filter method.

References

1. Timothy J Rudge, James R Brown, Fernan Federici, Neil Dalchau, Andrew Phillips, James W Ajioka, and Jim Haseloff. Characterization of intrinsic properties of promoters. *ACS synthetic biology*, 5(1):89–98, 2016.
2. Stefan Klumpp, Zhongge Zhang, and Terence Hwa. Growth rate-dependent global effects on gene expression in bacteria. *Cell*, 139(7):1366–1375, 2009.
